# Selective D3 receptor antagonism modulates neural response during negative emotional processing in substance dependence

**DOI:** 10.3389/fpsyt.2022.998844

**Published:** 2022-10-19

**Authors:** Ioanna A. Vamvakopoulou, Leon Fonville, Alexandra Hayes, John McGonigle, Rebecca Elliott, Karen D. Ersche, Remy Flechais, Csaba Orban, Anna Murphy, Dana G. Smith, John Suckling, Eleanor M. Taylor, Bill Deakin, Trevor W. Robbins, David J. Nutt, Anne R. Lingford-Hughes, Louise M. Paterson

**Affiliations:** ^1^Division of Psychiatry, Department of Brain Sciences, Imperial College London, London, United Kingdom; ^2^Neuroscience and Psychiatry Unit, Institute of Brain, Behaviour and Mental Health, The University of Manchester, Manchester, United Kingdom; ^3^Behavioural and Clinical Neuroscience Institute, University of Cambridge, Cambridge, United Kingdom; ^4^Department of Psychiatry, University of Cambridge, Cambridge, United Kingdom; ^5^Department of Psychology, University of Cambridge, Cambridge, United Kingdom

**Keywords:** emotional processing, dopamine, fMRI, D3 receptor, addiction, alcohol, polydrug, polysubstance

## Abstract

**Introduction:**

Negative affective states contribute to the chronic-relapsing nature of addiction. Mesolimbic dopamine D3 receptors are well placed to modulate emotion and are dysregulated in substance dependence. Selective antagonists might restore dopaminergic hypofunction, thus representing a potential treatment target. We investigated the effects of selective D3 antagonist, GSK598809, on the neural response to negative emotional processing in substance dependent individuals and healthy controls.

**Methodology:**

Functional MRI BOLD response was assessed during an evocative image task, 2 h following acute administration of GSK598809 (60 mg) or placebo in a multi-site, double-blind, pseudo-randomised, cross-over design. Abstinent drug dependent individuals (DD, *n* = 36) comprising alcohol-only (AO, *n* = 19) and cocaine-alcohol polydrug (PD, *n* = 17) groups, and matched controls (*n* = 32) were presented with aversive and neutral images in a block design (contrast of interest: aversive > neutral). Whole-brain mixed-effects and *a priori* ROI analyses tested for group and drug effects, with identical models exploring subgroup effects.

**Results:**

No group differences in task-related BOLD signal were identified between DD and controls. However, subgroup analysis revealed greater amygdala/insular BOLD signal in PD compared with AO groups. Following drug administration, GSK598809 increased BOLD response across HC and DD groups in thalamus, caudate, putamen, and pallidum, and reduced BOLD response in insular and opercular cortices relative to placebo. Multivariate analyses in *a priori* ROIs revealed differential effects of D3 antagonism according to subgroup in substantia nigra; GSK598809 increased BOLD response in AO and decreased response in PD groups.

**Conclusion:**

Acute GSK598809 modulates the BOLD response to aversive image processing, providing evidence that D3 antagonism may impact emotional regulation. Enhanced BOLD response within D3-rich mesolimbic regions is consistent with its pharmacology and with attenuation of substance-related hypodopaminergic function. However, the lack of group differences in task-related BOLD response and the non-specific effect of GSK598809 between groups makes it difficult to ascertain whether D3 antagonism is likely to be normalising or restorative in our abstinent populations. The suggestion of differential D3 modulation between AO and PD subgroups is intriguing, raising the possibility of divergent treatment responses. Further study is needed to determine whether D3 antagonism should be recommended as a treatment target in substance dependence.

## Introduction

Addiction poses a considerable burden to the individual and to society, and whilst we have made great strides in understanding the brain processes that lead individuals exposed to drugs to become dependent, the mechanisms which drive them to suffer repeated relapse, despite successful detoxifications and periods of abstinence remain unclear. After decades of research, we have few adequate treatment tools to support recovery, particularly in abstinence for relapse prevention. Recurrent relapses pose the greatest clinical challenge to addiction (1), so it is urgent to understand the underlying mechanisms, and find novel and successful therapeutic approaches.

Addiction is characterised by a shift from controlled consumption to compulsive drug use that occurs *via* neuroadaptive processes, which are exacerbated and maintained by the presence and/or emergence of negative affective states. Classically referred to as the 3-process model, the development of addiction involves binge/intoxication, withdrawal/negative affect and preoccupation/anticipation (2, 3). Acute withdrawal leads to the emergence of negative aversive states including anxiety, dysphoria, irritability, and higher levels of stress reactivity, capable of eliciting craving. This drives negative reinforcement and thus becomes a motivating factor in continued drug use and vulnerability to relapse (4–8). Chronic use and dependence can also lead to neuroadaptations in affective processing of both drug and natural reinforcers: these can result in dysregulation within the reward and incentive motivational systems of the brain (9) that serve to facilitate the development of chronic negative affective states. This leads to maladaptive behaviors and poor decision making, thus perpetuating the chronicity of substance use disorders and the enduring vulnerability to relapse, despite abstinence. The development of such aversive emotional states has been described as the “dark side” of addiction (3, 10).

Substance dependence is also associated with difficulty in regulating emotions. Growing evidence shows alterations in neural processing of emotional stimuli, with notable dysregulation in corticolimbic regions including amygdala, insula and ventromedial, medial prefrontal (PFC) and rostral anterior cingulate cortex (ACC) (11, 12). Studies investigating the neural response to negative emotional stimuli typically measure the response to negatively valent faces or aversive imagery, sometimes incorporating stress-sensitivity paradigms. Findings are mixed, but a number of studies in alcohol and cocaine dependence report increased neural response within limbic regions under evocation of negative emotional states (13–17) and blunted response within prefrontal regions associated with emotional regulation including ventromedial and medial prefrontal cortex (PFC) and rostral anterior cingulate cortex (ACC) (15, 17–20) relative to controls. These regions are also involved in the appraisal and expression of negative emotions (21), which might suggest an imbalance in top-down control of emergent emotional states. Other studies support the idea of aberrant emotional processing in substance dependence (8, 14, 22–24). Taken together, this may be indicative of a failure of cognitive control in managing emergent emotional responses to stress, trauma and emotive situations, which could prove particularly detrimental to recovery during abstinence. This is consistent with the idea of compromised prefrontal cortical capacity leading to inadequate decision making when confronted by maladaptive or inappropriate limbic responses to emotionally valent stimuli (25–27).

Distinct profiles of response to emotional processing may arise according to the drug of dependence (9). In general, alcohol dependence is more robustly associated with dampening of BOLD responses in fronto-cortical regions across a range of emotional regulation tasks (15, 17, 28–30). In cocaine dependence, similar reductions in cortical response have been observed although fewer studies are available (18, 22, 23). Findings within limbic regions are more mixed. For example increases in amygdala activation have been observed in alcoholism (13, 15), and in some (14, 20), but not all studies in cocaine users (22).

Differences in response may also arise due to differences in stress vulnerability, exposure to trauma and trait characteristics such as anxiety and depression, with amygdala hyperreactivity commonly described in response to negative emotional stimuli (31–34). Since negative affect represents a risk factor for the development of addiction, and anxiety, depression and childhood adversity are highly co-morbid with addiction, sub-groups of stress-sensitive individuals may exist within substance dependent cohorts with implications for treatment. Trauma and neglect in childhood in particular is associated with worse emotional dysfunction (35), and is highly prevalent in illicit/polydrug dependence (36, 37).

There is a wealth of evidence linking dopaminergic dysfunction to substance abuse and dependence generally (38), particularly in relation to its well-documented role in reward and motivational processing (39). But decades of endeavor particularly in relation to D2 receptor function has failed to produce new efficacious treatments. The role of dopaminergic function in emotional regulation and its contribution to impaired emotional processing in substance dependence is unclear (40). Acute stress is known to increase dopamine release in the accumbens and prefrontal cortex (41), which may negatively impact addicted individuals with compromised dopaminergic function and/or negative affect (42). Emotional processing is dependent on the aforementioned corticolimbic brain structures that are subject to dopaminergic innervation, making them good candidates for targeted pharmacological therapeutic approaches.

The potential for modulation of emotional processing by the dopamine D3 receptor (D3R) has yet to be fully explored, despite the potential clinical utility of selective antagonists in brain disorders (43). D3 receptors are found at highest density in the mesolimbic dopaminergic system and ventral forebrain, particularly nucleus accumbens, substantia nigra, pallidum and thalamus (44–46), so are well placed as potential mediators of emotions, reward, motivation and stress reactivity, and by extension have a likely mechanistic role in drug seeking behavior and relapse (47, 48). Although less abundant than D1 or D2 receptors, D3 receptors have higher binding affinity for dopamine, such that small changes in receptor density or function can lead to dramatic changes in neurotransmission, and thus may be essential for normal dopaminergic activity (39, 43, 49, 50). It has been speculated that dopamine autoreceptor action might contribute to the inhibition of phasic dopaminergic reward signaling by tonic extracellular dopamine. Thus, an increase of dopamine *via* the inhibition of D3 autoreceptors could be a useful pharmacological therapeutic approach in substance dependence (38, 51). Indeed, greater levels of D3 receptors have been robustly observed in substance dependence in PET and post-mortem studies (47, 52–56). Preclinical evidence has suggested a causal link between exposure to dopamine elevating drugs including cocaine, alcohol, and nicotine and increased D3 receptor expression, possibly attributed to plasticity changes in response to chronic drug use (57–60).

Selective D3 antagonism may therefore represent a new therapeutic approach, and preclinical and clinical evidence supports this. Selective D3 antagonists have been shown to reverse the acquisition and expression of drug-seeking behavior, to attenuate cue-, drug-, and stress-induced relapse and to reduce self-administration and conditioned place preference in animal models of alcohol (60, 61), and in particular cocaine dependence (50, 62–69). There is a potential mechanistic role emerging for the basolateral amygdala in stress-related cocaine reinstatement (65). Clinically, acute doses of GSK598809, a D3 antagonist with > 100-fold selectivity for D3 > D2 receptors, appeared to partially alleviate nicotine craving in short term abstinent smokers (70), and reduced food-cue attentional bias in low restrained eaters (71), and to modulate anticipatory reward responding in D3-rich regions in substance dependence, in a manner indicative of clinical benefit (51). D3 antagonism may also attenuate cue-induced responses and reward impulsivity in healthy individuals (72), and ‘normalise’ reward-related responding in depression (73), although these studies used the non-selective D2/D3 antagonist amisulpride.

Whilst there is no direct evidence that D3 antagonism reduces stress or negative affect in humans *per se*, its efficacy in animal models of stress-induced drug-related behavior and of post-traumatic stress disorder (74, 75), coupled with its potential to modulate reward circuitry and reduce cigarette craving renders D3 a credible target for negative affect in addiction. Rather than altering the primary reinforcing properties of salient rewards (i.e., the drug itself), D3 antagonism may instead disrupt responsiveness to other relevant stimuli that reinforce drug-seeking behavior including environmental cues, or stress (48). We speculate that D3 antagonism could ameliorate the negative emotional state characterising addiction and that during abstinence, this could represent a target for relapse prevention.

We investigated the effect of an acute dose of the selective D3 receptor antagonist, GSK598809, on negative emotional processing using a functional MRI paradigm to assess the neural response to aversive vs. neutral images in abstinent substance dependent individuals (DD) compared with controls (HC), with a particular focus on individuals with alcohol and/or cocaine dependence. We conducted *a priori* ROI analyses in amygdala, nucleus accumbens (NAcc), ventral pallidum (VP), substantia nigra (SN) and medial prefrontal cortex (mPFC), based on their high D3 receptor density and/or importance in negative emotional processing, and also carried out exploratory whole brain analyses. Given the potential for differences according to substance of dependence and evidence that D3 antagonism might be more effective in certain subpopulations (e.g., psychostimulant users), we further investigated whether distinct subgroup differences in response exist by comparing alcohol-only (AO) with alcohol-cocaine polydrug dependent (PD) individuals.

Finally, given the comorbidity between substance dependence and other stress-vulnerability factors, we explored associations between BOLD signal change and clinical traits of anxiety and history of childhood trauma to determine whether these factors modulate the neural response to aversive processing or impact on response to drug. We hypothesised that heightened limbic and decreased frontal cortical BOLD signal would be observed in drug dependent relative to control groups during aversive vs. neutral stimuli. We hypothesised that D3 antagonism would normalise aberrant BOLD response in the drug dependent population only, and that healthy individuals would not display changes in brain activity following GSK598809 administration.

## Materials and methods

### Study design

Data were obtained from the Imperial College, Cambridge and Manchester (ICCAM) platform study (76). The design was a multi-center, double-blind, pseudo-randomised, placebo-controlled study and consisted of five visits: an initial baseline scan and four experimental scan sessions. Two experimental sessions are relevant to assess the effects of D3 antagonism on the neural response to evocative images in substance dependent and healthy individuals; the placebo and D3R antagonist GSK598809 sessions, at which a total of 87 individuals received the medication and completed the scan. Structural and functional magnetic resonance images (fMRI) were acquired 2 h following acute administration of the D3 selective antagonist, GSK598809 (60 mg) and placebo (Vitamin C), on two separate occasions separated by at least 5 days to allow for drug washout. The study was conducted in accordance with the Declaration of Helsinki, with ethical approval obtained from West London and Gene Therapy Advisory Committee National Research Ethics Service Committee (REC reference: 11/H0707/9).

Participants were recruited from local drug and alcohol clinical services, from healthy volunteer databases, *via* multimedia advertising and *via* word of mouth. Following an initial telephone interview to identify those who met basic inclusion criteria, participants were invited for a screening visit to provide written informed consent and assess eligibility. Participants attended a diagnostic interview with a clinician who determined the diagnoses of DSM-IV substance dependence. All substance dependence histories were subsequently reviewed by two psychiatrists to ensure uniformity of diagnostic thresholds across sites, and any discrepancies arbitrated by a third psychiatrist [see (76) for full study description]. All participants also provided an account of lifetime drug and alcohol history, along with demographic characterisation, and completed the Spielberger Trait Anxiety Index (STAI-T, (77), childhood trauma questionnaire (CTQ) and perceived stress scale (PSS) (78, 79). Eligible participants were invited back for experimental sessions, two of which involved an oral dose of the placebo vitamin C (100 mg, received on session two or three) and the D3R antagonist GSK598809 (60 mg, received on session four or five). A weighted randomisation was used to avoid the potential for loss of placebo data due to study drop-out (76).

### Participant characteristics

Inclusion criteria for the ICCAM study were those meeting DSM-IV criteria for current or prior alcohol, opioid or cocaine dependence, who were abstinent for at least 4 weeks prior to experimental assessments (median 9 months abstinent) (80). It was originally planned to recruit samples of individuals that were dependent on only one substance, but this was not possible to achieve. Whilst an alcohol-only dependent group emerged, those dependent on cocaine and/or opioids were most often polydrug dependent [see (76)]. In this analysis, those with a diagnosis of alcohol dependence only (AO subgroup), and those with alcohol plus cocaine dependence (PD subgroup) were considered. In the alcohol-only group, nicotine dependence (current or historic) was permitted but otherwise this was a ‘clean’ sample of alcohol dependent individuals. In the PD group, other lifetime co-dependencies in addition to alcohol and cocaine were permitted e.g. nicotine, opiates, benzodiazepines etc., and thus co-dependence was common in our PD sample, and was representative of the UK polydrug dependent population; all lifetime/co-dependencies are listed in Table 1. Participants were required to be abstinent from all drugs of dependence, except nicotine, for at least 4 weeks prior to the experimental sessions. Healthy controls were recruited who had never met substance dependence criteria (excluding nicotine) and were matched where possible for age, sex, and nicotine smoking status. We were thus able to compare responses between a ‘clean’ alcohol-only dependent group and a polydrug group who had alcohol *and* cocaine dependence in common, relative to controls who were matched for nicotine dependence. All participants were able to read, comprehend and record information in English. Exclusion criteria included current, regular use of psychoactive medication (including antipsychotics, antidepressants, or anticonvulsants), current primary axis I diagnosis, history of psychosis or severe mental illness (history of depression or anxiety were permitted given their high comorbidity), MRI contraindications, pregnancy, history or presence of severe neurological disorder and clinically significant head injury. Current intoxication was excluded, including recent drug use, confirmed by negative breath alcohol and urine drugs of abuse screen (including cocaine, opiates, methadone, benzodiazepines, amphetamines, barbiturates). Participants were also requested to refrain from cannabis use for at least seven days prior to each session but positive urine test results for cannabinoids were permitted given the long half-life of cannabinoid metabolites.

**TABLE 1:** Sample characteristics for each group.

*Sample characteristics*						
Demographics	Healthy controls (*n* = 32)	Drug dependent (*n* = 36)	Group comparisons^a^	PD (*n* = 17)	AO (*n* = 19)	Group comparisons^b^
Age (years; mean, SD)	41.9 ± 8.8	43.1 ± 8.6	*t*_(66)_ = –0.586, *p* = 0.560	41.1 ± 7.9	44.9 ± 8.9	*F*_(2,67)_ = 1.032, *p* = 0.362
Age range (years)	25–64	30–60		30–58	30–60	
Gender ratio (M:F,%)	25:7, 78	27:9, 75	χ^2^_(1)_ = 0.092, *p* = 0.762	12:5, 71	15:4, 79	χ^2^_(2)_ = 0.44, *p* = 0.802
Site of scan (Imperial:Camb:Man)	11:12:9	19:10:7	χ^2^_(2)_ = 2.34, *p* = 0.311	10:4:3	9:6:4	χ^2^_(4)_ = 2.83, *p* = 0.587
Years of education (median, IQR)	12.5 ± 5.0	11.0 ± 1.0	*U* = 394.0, *p* = 0.015	11.0 ± 1.0	11.0 ± 2.0	*H*_(2)_ = 8.275, *p* = 0.016 *HC > PD
Drug and Alcohol Measures						
Lifetime alcohol dependence n (%)	0	36 (100)		17 (100)	19 (100)	
Lifetime cocaine dependence n (%)	0	17(47.2)		17 (100)	0	
Lifetime opiate dependence n (%)	0	10 (27.8)		10 (59)	0	
Lifetime nicotine dependence n (%)	20 (63)	32 (89)	χ^2^_(1)_ = 6.56, *p* = 0.010	16 (94)	16 (84)	χ^2^_(2)_ = 7.05, *p* = 0.030
Current smokers *n*, (%)	18 (56.3)	27 (75)	χ^2^_(1)_ = 2.66, *p* = 0.103	13 (77)	14 (74)	χ^2^_(2)_ = 2.69, *p* = 0.260
Nicotine exposure (pack/year; median, IQR)	4.4 ± 22.1	22.1 ± 21.3	*U* = 812.5, *p* = 0.004	24.0 ± 27.4	20.0 ± 8.0	*H*_(2)_ = 8.768, *p* = 0.012 *PD > HC, *AO > HC
Months abstinence (median, IQR)		9.0 ± 20.9		11.0 ± 21.3	8.0 ± 20.5	*U* = 140.5, *p* = 0.510
Months abstinence (range)		1–102		1–102	1–79	
Mood measures						
STAI-T Scores (mean, SD) CTQ Scores (median, IQR)	28.8 ± 6.5 32.0 ± 7.8	42.2 ± 11.8 43.5 ± 28.5	*t*_(66)_ = –5.685, *p* < 0.001 *U* = 793.5, *p* = 0.007	43.1 ± 11.1 53.0 ± 26.0	41.4 ± 12.7 36.0 ± 18.5	*F*_(2,67)_ = 16.127, *p* < 0.001 *PD > HC, *AO > HC χ^2^_(2)_ = 14.319, *p* < 0.001 *PD > HC, *PD > AO
PSS Scores (mean, SD)	15.3 ± 6.2	21.1 ± 7.9	*t*_(66)_ = –3.350, *p* = 0.001	23.1 ± 6.1	19.3 ± 9.1	*F*_(2,67)_ = 6.989, *p* = 0.002 *PD > HC

Data are presented as mean ± standard deviation (SD) for normally distributed data and as median ± interquartile range (IQR) for non-normally distributed data. Chi squared (χ^2^) tests were used for categorical variables. ^a^Two-group comparisons were conducted between HC and DD using Student’s *t*-test (*t*) or Mann–Whitney-*U* tests (U) as applicable. ^b^Three-group comparisons were conducted between HC, PD, and AO groups using ANOVA (F) or Kruskall–Wallis tests with Bonferroni *post hoc* comparisons as applicable, significance level *P* < 0.05. *Indicates where *post hoc* testing revealed significant 2-group differences. ^c^Indicates months abstinent from all drugs of dependence. STAI-T, Spielberger Trait Anxiety Index; CTQ, childhood trauma questionnaire; PSS, perceived stress scale.

#### Analysed sample

Of the 87 individuals receiving study medication, 73 met the criteria of alcohol-only or alcohol-cocaine polydrug dependence. We excluded an additional five participants due to poor demographic matching (*n* = 1), signal drop-out (*n* = 1) and excessive motion in the scanner (*n* = 3), defined as > 90 extremes of movement (> 0.5 mm) during both placebo and GSK598809 scans in at least one run of the task. Thus, a total of 68 participants were included in the analysis: healthy controls (*n* = 32) and substance dependent (*n* = 36), comprising alcohol-cocaine dependent (*n* = 17) and alcohol-only (*n* = 19) groups.

### Image acquisition and pre-processing

Detailed structural and functional acquisition procedures and pre-processing and task modeling steps can be found in (81). The Imperial College London and Cambridge University centers utilised identical 3T Siemens Tim Trio systems, and Manchester University operated a 3T Philips Achieva scanner. For structural imaging, all sites acquired T1-weighted volumes for registration purposes, using a magnetisation-prepared rapid gradient echo (MPRAGE) sequence that were harmonized across sites. In London and Cambridge the parameters were as follows: TR = 2300 ms, TE = 2.98 ms, TI = 900 ms, flip angle = 9°, field of view = 256 mm, image matrix = 240 × 256 with a resolution of 1 mm isotropic, and in Manchester were: TR = 6.8 ms, TE = 3.1 ms, TI = 900 ms, flip angle = 9°, field of view = 270 mm, image matrix = 256 × 256 with an in-plane resolution of 1.055 × 1.055 mm and a slice thickness of 1.200 mm.

For functional acquisition, all three centers adopted a harmonised multi-echo gradient echo echoplanar imaging sequence as follows (TR = 2000 ms, TE = 13 ms and 31 ms, flip angle = 80°, field of view = 225 mm, image matrix = 64 × 64, in-plane resolution = 3.516 × 3.516 mm, slice thickness = 3.000 mm. 36 abutting oblique axial slices were collected (34 in Manchester) for each volume, in an ascending manner at a ∼30° angle to the anterior commissure-posterior commissure line.

A combination of neuroimaging tools were used to pre-process structural and functional images [Analysis of Functional NeuroImages (AFNI), FreeSurfer, Advanced Normalization Tools (ANTs), and FMRIB Software Library’s (FSL)], according to (81). T1 images were corrected for image intensity, extracerebral tissue was removed, and warping to MNI152 space. Functional images underwent slice-time correction, realignment, co-registration (using a boundary-based approach) to the T1-weighted image and were subsequently warped to 2 mm MNI152 space. Finally, smoothing was performed using a three-dimensional Gaussian kernel of full width at half maximum of 6.0 mm.

### Evocative image task

#### Task description

Participants performed the Evocative Image Task (16, 81) to measure the neural response to processing of emotionally valent aversive stimuli. To minimise learning effects, participants first familiarised themselves with the task (using different images to those used in the actual task) outside the scanner before each session. During the scan, participants were presented with aversive images of injury or threat and neutral images of animate and inanimate objects provided by the International Affective Picture System (IAPS) library^1^. Each image in this library was independently rated for arousal and valence (pleasure), and affective norms calculated across multiple studies. The images selected for this task had no overlap between valence scores for neutral and aversive stimuli, and only minimal overlap between arousal scores. Valence scores ranged from 4.03 to 6.58 for neural and 1.31 to 3.77 for aversive images (all less than the neutral mid-point of 5), and arousal scores ranged from 1.72 to 5.85 for neutral and 4.34 to 7.35 for aversive images. Mean (±SD range) scores for valence and arousal were equal across sessions and scores were as follows: 5.17 ± 0.54–0.72 for neutral and 2.53 ± 0.58–0.69 for aversive. Mean scores for arousal across sessions were 3.47 ± 0.79–0.9 for neutral and 5.95 ± 0.71–0.85 for aversive. No images containing drugs, alcohol or food were presented, to avoid cue-induced reactivity. The task and valence/arousal calculations are available upon request.

The task was delivered in a block design comprising two runs. Each run contained eight blocks (four neutral and four aversive) of six images presented in pseudo-randomised order, with each neutral block always followed by an aversive block. Each image was presented for 5 s, followed by a 400 ms inter-stimulus interval (fixation cross) such that the total block length was 32.4 s. At the beginning of each run, a fixation cross was presented for 12 s and each block was separated by a 15 s rest period to prevent any carry-over effects, such that each run had a total length of 6 min 32 s, and 196 volumes were collected. The second run of the task was identical except that the images within blocks were presented in a different order, and the order of block presentation was shuffled (although aversive still followed neutral blocks). Across each of the two experimental sessions, different images were presented to avoid any habituation effects. Thus, 96 unique pictures were presented in total; 48 neutral and 48 aversive images. Images were counterbalanced for valence and arousal scores between blocks and across the experimental visits. Participants were required to press their response pad once per image to ensure they were paying attention.

#### Functional MRI task modelling

Task modeling and lower-level analyses were performed according to (81), using tools from FMRIB Software Library’s (FSL version 5.0.6) and FMRI Expert Analysis Tool (FEAT) (version 6.00). Two explanatory variables were used: one for aversive and one for neutral images which were modeled as blocks lasting 32.4 s in a general linear model. The contrast of interest was that of aversive vs. neutral images (aversive > neutral), with greater limbic BOLD response expected in response to aversive stimuli compared with neutral.

#### Lower-level (single subject) analyses

First, each block was modeled as a boxcar function and convolved with the hemodynamic response function. Six motion parameters were added to the model as confounding regressors, and a high-pass filter with a cut-off of 100 s was applied to remove low frequency artifacts. This created two maps for each individual; one for each run of the task. Next, a fixed-effects analysis was conducted to obtain the mean BOLD signal across the two runs for each participant at each of the placebo and D3 imaging sessions. Finally, a fixed effects analysis was conducted to subtract the mean placebo from the mean GSK598809 lower-level data to create a drug difference image (GSK598809 – placebo) for each individual, such that positive BOLD signal change represented a greater response to GSK598809, and negative values represented a greater response to placebo.

#### Group level analysis

Using FMRIB’s Local Analysis of Mixed Effects 1 (FLAME-1, version 6.0.1) whole brain analyses were conducted to investigate the effect of group on negative emotional processing (HC compared with DD and PD compared with AO). Due to the multi-scanner nature of the study all analyses controlled for scanning center, given the possibility for inter-center differences (81). Between-group effects were analysed using an unpaired t-test, controlling for age and center, for the aversive > neutral contrast, using data collected at the placebo session only. The effect of GSK598809 relative to placebo between groups (GSK598809 minus placebo) was investigated in a separate unpaired *t*-test, also controlling for age and center. Cluster-based Z statistical images were thresholded at Z > 2.3 (*p* < 0.05, corrected). Local maxima co-ordinates within significant clusters were defined according to the Harvard-Oxford cortical and subcortical structural atlases. Contrast of parameter estimates (COPEs) were extracted from the activated clusters of interest for graphical display as appropriate, using FSL’s Featquery and converted to mean percentage BOLD signal change. As the scope of our study was to investigate the modulation of dopaminergic signaling relevant to emotional processing, clusters from the occipital cortex were not examined further.

#### Region of interest analysis

*A priori* regions of interest (ROIs) were chosen according to their close association with negative emotional processing and high D3 receptor expression. The ventral striatum (NAcc), ventral pallidum (VP) and substantia nigra (SN) were selected based on our previous publication (51) in which D3 antagonism was shown to modulate monetary reward processing. These regions were have high D3 receptor expression levels according to PET studies (46). In addition, the bilateral amygdala and mPFC were chosen due to their association with aversive and negative emotional processing. Further details on ROI generation can be found in Supplementary Figure 1. Using FSL’s Featquery, COPEs for the contrast of aversive > neutral were extracted from the *a priori* masks using data from the single-subject level outputs at the placebo and D3 imaging sessions, and converted into percentage BOLD signal change.

### Statistical analysis

#### Demographics and region of interest analysis

Analyses were conducted using SPSS version 27. For demographic data, normality was assessed using Shapiro–Wilk tests. Group differences were investigated using unpaired *t*-tests or Mann–Whitney *U* tests for two group comparisons, and one-way ANOVAs or Kruskal–Wallis tests for three group comparisons for normally and non-normally distributed data respectively. *Post hoc* testing was conducted using Bonferroni correction, as appropriate. Chi-squared tests were conducted for categorical variables. To investigate the effect of drug, group, and drug*group interaction in the ROIs of interest, multivariate analysis of covariance (MANCOVA) was conducted with drug (placebo, D3) and ROI as within-subjects factors and group (HC, DD) or subgroup (AO vs. PD) as between-subjects factor, controlling for center and age. Significant overall effects as determined by Pillai’s trace were further explored using a 2 × 2 mixed analysis of variance (ANOVA) in the relevant ROI, with drug (placebo, D3) as the within-subject factor and subgroup (PD, AO) as the between-subject factor, while controlling for age and center.

#### Correlation/regression analysis

Exploratory associations were investigated between BOLD signal response and trait measures (anxiety, childhood trauma) in the ROIs of interest, to determine whether there was any impact of the experience of childhood trauma or trait anxiety on response to the task (under placebo only, aversive > neutral contrast) and whether trait anxiety or childhood trauma had any impact on response to drug (D3 minus placebo contrast) using two-way linear regression models available in R (82). Associations were tested across all individuals, controlling for group. Twenty regression models were conducted in total; each of five ROIs was tested investigating associations between (1) BOLD signal response to task and childhood adversity (CTQ score), (2) BOLD signal response to task and trait anxiety (STAI-T score), (3) BOLD signal response to drug and CTQ score, and (4) BOLD signal response to drug and STAI-T score. The left and right substantia nigra and pallidum ROIs were entered into the same model with hemisphere included as control variable, to account for the repeated measure across L and R hemispheres in these regions. Trait by group interaction terms were also included in each model to determine whether a model including between-group differences in the associations could better explain the data, i.e., whether the label of substance dependence might moderate the association between dependent variables. It was found that including the interaction terms did not improve the models (no improvement in standard error of residuals nor the adjust *R* squared value, however, they did increase the standard error of the estimates) so these were not further considered. FDR correction was applied for correction of multiple comparisons; corrections were performed across model predictors rather than the model as a whole. Bonferroni correction was then additionally applied to determine which comparisons survived the 20 tests conducted [significance level *p* = 0.0025 (0.05/20)].

## Results

### Sample characteristics

Participants were well matched between HC and DD and between HC, PD, and AO groups for age, gender and site distribution (Table 1). Significant group differences were observed in years of education with DD completing fewer years of education, which was primarily driven by the PD subgroup. Whilst the proportion of current smokers did not differ between groups, there was a higher incidence of lifetime nicotine dependence and nicotine exposure in the DD group relative to controls. Both substance dependent subgroups exhibited higher trait anxiety as compared with controls but there was no difference between AO and PD groups on STAI-T scores (*t* = 0.438, *p* = 0.664). Substance dependent individuals also experienced higher childhood trauma (CTQ) and perceived stress scale scores relative to controls, driven primarily by the PD group, which presented with significantly higher scores than both AO and HC individuals [χ^2^_(2)_ = 14.319, *p* = 0.001 and *F*_(2_,_67)_ = 6.989, *p* = 0.002 respectively].

### BOLD response to evocative task in substance dependence and controls

#### Task related BOLD response to aversive image processing

**FIGURE 1:**
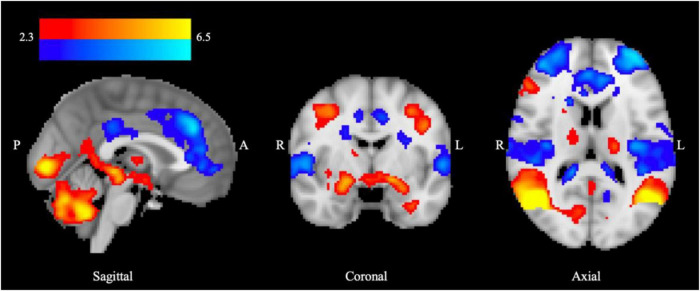
BOLD response to aversive processing in all groups combined following placebo administration. Increased limbic BOLD signal across HD and DD groups for the aversive > neutral contrast is denoted in red, and decreased fronto-cortical BOLD signal indicated in blue. Color bar shows *z*-stat values representing average BOLD signal change across groups using FSL’s FLAME-1, mixed-effects analysis (*n* = 68, Z > 2.3, *P* < 0.05), controlling for center and age. MNI coordinates: *x* = 0.5 (sagittal), *y* = –4.7 (coronal), *z* = 13.7 (axial).

A whole brain analysis investigating the response to aversive compared with neutral image processing (aversive-neutral contrast) during the EIT task under placebo condition across all individuals revealed greater BOLD response in three clusters (Figure 1, red-yellow palate) including bilateral amygdala, thalamus, globus pallidus, hippocampus, left putamen and right insular cortex, visual cortex, and cerebellum. By contrast, lower BOLD signal was observed in six clusters (Figure 1, blue palate), including cingulate gyrus, bilateral middle temporal gyrus, ACC, frontal pole, and portions of the mPFC. Whole brain activation maps are shown in Supplementary Figure 2, alongside cluster peak activations (Supplementary Table 1).

#### Increased BOLD response in polydrug relative to alcohol-only subgroups under placebo

**FIGURE 2:**
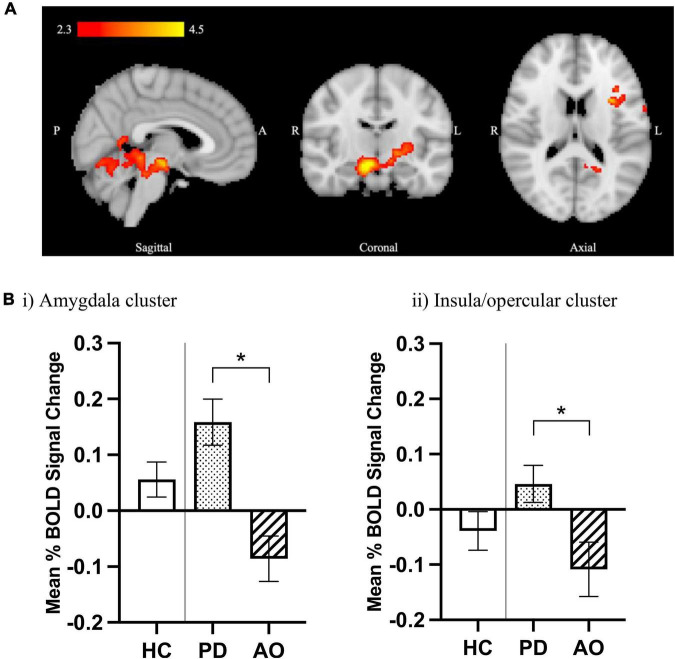
Increased limbic BOLD response to aversive processing in PD relative to AO groups under placebo. **(A)** Increased BOLD signal in the PD > AO group for the aversive > neutral contrast. Color bar shows *z*-stat values from an unpaired *t*-test using FSL FLAME-1, mixed-effects analysis (*Z* > 2.3, *p* < 0.05), controlling for center and age. MNI coordinates: *x* = 3.6, *y* = –13.7, *z* = 16.9. **(B)** Mean% BOLD signal change from extracted significant clusters in (i) cluster 1 including amygdala, putamen, hippocampus, left thalamus, cingulate gyrus, and pallidum and (ii) cluster 2 including left insula, left precentral gyrus, left postcentral gyrus, left central opercular cortex, and left inferior frontal gyrus (pars opercularis). Data are mean ± SEM for PD (*n* = 17) and AO (*n* = 19) groups, plotted next to HCs (*n* = 32) for visual reference. *Indicates significant difference as determined by whole brain analysis in **(A)**.

When BOLD responses were compared between HC and DD groups for the contrast of aversive > neutral stimuli no significant differences were found in whole brain analyses (threshold Z > 2.3) or in the *a priori* ROIs (amygdala, NAcc, mPFC, VP, SN) (Supplementary Table 2). However, significant differences were identified between the PD and AO subgroups, with greater BOLD response observed in the PD relative to the AO group. In the whole brain analysis, increased BOLD response was observed in two significant clusters (Figure 2A), the first including areas of the amygdala, putamen, hippocampus, left thalamus, cingulate gyrus, and pallidum (cluster 1) and the second within left insula, left precentral and postcentral gyrus, left central opercular cortex, and left inferior frontal gyrus (pars opercularis) (cluster 2). Data were extracted from each cluster for both PD and AO groups and plotted alongside HC participants for visual reference (Figure 2B). Peak activation co-ordinates are provided in Supplementary Table 3. In the *a priori* ROIs the same pattern was observed whereby lower BOLD signal change was observed across ROIs in the AO relative to PD group in response to placebo, although the effect of subgroup overall did not reach significance [*F*_(1_,_31)_ 0.730, *p* = 0.399, Supplementary Table 4]. Despite some level of anatomical overlap between the amygdala ROI and the extracted amygdala cluster, no significant between-subgroup difference was observed within the amygdala ROI.

### Effect of D3 antagonism on aversive processing in control and substance dependent groups

#### Whole brain analyses

**FIGURE 3:**
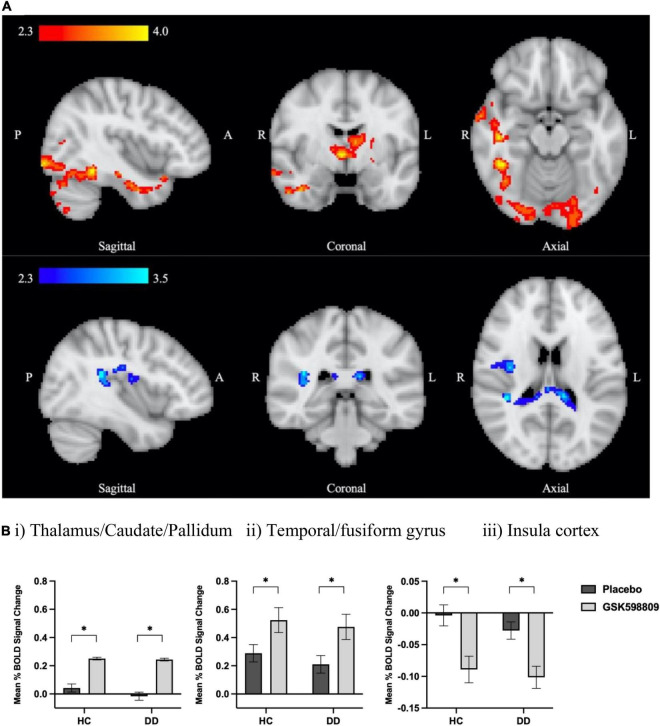
BOLD signal change following D3 receptor antagonism in response to aversive image processing. **(A)** Average BOLD signal change in aversive > neutral contrast following D3 antagonism relative to placebo (inputs were drug difference images for GSK598809-placebo) derived from a whole brain analysis using FSL’s FLAME-1 in HC and DD groups (*n* = 68). Color bars show *z*-stat values (threshold Z > 2.3, *P* < 0.05 corrected). Red colors indicate increased BOLD signal for the effect of drug (GSK598809 – placebo, MNI coordinates: *x* = 41.9, *y* = –4.7, *z* = –16.5), and blue colors indicate decreased BOLD signal (MNI coordinates: *x* = 39.2, *y* = –34.4, *z* = 18.2). **(B)** BOLD signal change in extracted clusters in HC (*n* = 32) and DD (*n* = 36) groups in (i) thalamic/caudate/pallidum cluster, (ii) temporal/fusiform gyrus cluster, and (iii) insula cluster. NB: there is no overlap between this insula cluster and that found to be altered in the polydrug versus alcohol-only subgroup comparison in Figure 2; they were lateralised to right and left hemispheres respectively. Data are mean ± SEM. *Indicates significant difference as determined by whole brain analysis in **(A)**.

An overall drug effect was observed in both HC and DD groups relative to placebo, with significantly increased BOLD signal identified following GSK598809 administration in three clusters, occupying regions including bilateral thalamus, left caudate, and right pallidum (cluster 2) occipital/visual cortex/cerebellum (cluster 1), and middle/inferior temporal, fusiform and parahippocampal cortex (cluster 3) (Figure 3, red palate). Decreases in BOLD signal were observed in a single cluster in response to GSK598809, occupying the central and parietal opercular and right insular cortices (Figure 3, blue palate). Peak cluster co-ordinates are provided in Supplementary Table 5. No significant between group differences were observed in response to GSK598809 (i.e., no drug*group interactions were observed in HC compared with DD, or AO vs. PD subgroup analyses).

#### Region of interest analysis

**FIGURE 4:**
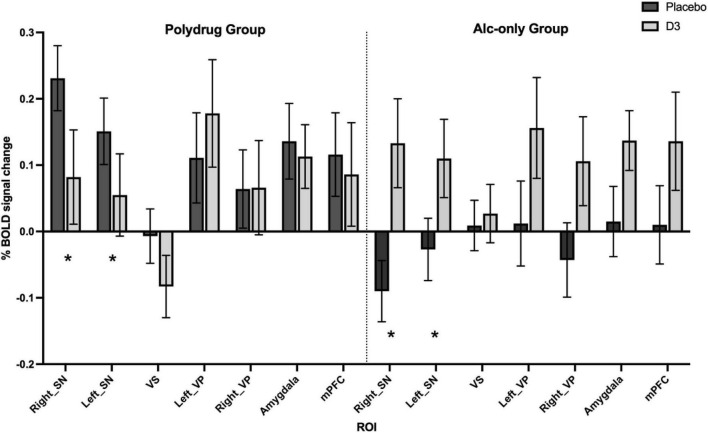
Differential effect of D3 antagonism by ROI in AO vs. PD groups. A significant subgroup by drug by ROI interaction was observed in multivariate analysis [Pillai’s trace; *F*_(6_,_26)_ = 2.659, *p* = 0.038], with *post hoc* analysis revealing significant effects in right and left substantia nigra (SN), see Supplementary Table 5. *Denotes the regions in which significant interactions occurred, primarily driven by a divergent effect of drug in AO compared with PD subgroups. Data are % BOLD signal change during the aversive > neutral contrast following administration of placebo and GSK598809 across PD (*n* = 17) and AO (*n* = 19) groups, controlling for age and center. Values are estimated marginal means (±SEM, with covariates appearing in the model evaluated at the following values: Centre1 = 0.11, Centre2 = 0.31, Age = 43.11).

No significant drug, group, or interaction effects were observed in the aversive > neutral contrast within the *a priori* ROIs in the HC vs. DD comparison (see Supplementary Table 2 and Supplementary Figure 2A). However the response to drug broadly mirrored that of the whole brain analysis whereby, when taking all participants together, an increased response to D3 antagonism was observed in all ROIs except the ventral striatum in which there was no change [significant drug*ROI effect; *F*_(6_,_26)_ = 2.696, *p* = 0.036, Supplementary Figure 3 and Supplementary Table 4]. By contrast, differences in response to drug were revealed when comparing AO and PD subgroups (Figure 4) whereby a significant drug*subgroup interaction was observed with an increase in BOLD response to drug evident in AO relative to PD subgroups [*F*_(1_,_31)_ = 6.591, *p* = 0.015, Supplementary Figure 2B], suggesting a differential effect of D3 antagonism on ROI BOLD signal in AO vs. PD subgroups. Furthermore, a subgroup*drug*ROI interaction was also observed [*F*_(6_,_26)_ = 2.659, *p* = 0.038] suggesting the D3 antagonism by subgroup effect varies according to ROI (Figure 4 and Supplementary Figure 4). Exploration of this interaction showed that the differential response to drug emerged due to divergent responses in the D3-rich substantia nigra regions whereby BOLD response to drug in the AO group was higher relative to the PD group where there was a decreased response to drug [Figure 4; significant group*subgroup interaction in right and left SN; *F*_(1_,_31)_ = 17.5, *P* < 0.001, and *F*_(1_,_31)_ = 6.365, *P* = 0.017 respectively].

### Regression analyses; relationship between BOLD response and childhood adversity and trait anxiety

The association between trait anxiety and experience of childhood trauma with BOLD response to aversive processing under placebo and in response to drug was investigated using regression analyses for each ROI. After multiple comparison correction, linear regression models revealed weak positive associations (low β coefficients) between childhood adversity and BOLD signal in the SN in response to task (aversive > neutral contrast, *t* = 2.65, *p*_corr_ = 0.045) and between trait anxiety and BOLD signal in the VP in response to task (*t* = 3.11, *p*_corr_ = 0.011, see Supplementary Table 6 and Supplementary Figure 5). These associations did not survive additional Bonferroni correction for multiple comparisons.

When investigating the impact of childhood adversity and trait anxiety in response to D3 antagonism, weak negative associations were observed between BOLD response and childhood adversity in the SN [*t* = –3.70, *p*_corr_ = 0.0016, *p*_BonferroniCorr_ = 0.032] and between BOLD response and trait anxiety in the VP (*t* = –3.43, *p*_corr_ = 0.004) and SN (*t* = –2.56, *p*_corr_ = 0.029), see Supplementary Table 7 and Supplementary Figure 6. Only the negative association between BOLD response in the SN and childhood adversity survived additional multiple comparison correction.

## Discussion

We investigated the effect of the dopamine D3 receptor antagonist GSK598809 on negative emotional processing evoked by aversive non-drug and alcohol-related images in abstinent cocaine-alcohol polydrug and alcohol-only dependent individuals compared with controls. To our knowledge, this is the first demonstration of the effects of selective D3 receptor antagonism on negative emotional processing in substance dependence in man. Importantly, the dependent ‘real-world’ UK sample studied was representative of those for whom relapse prevention treatment might prove most beneficial. We found patterns of task-related BOLD activation in the aversive vs. neutral contrast, that were in accordance with the expected neural substrates of negative emotional processing. In response to the D3 antagonist, we found greater BOLD response following acute GSK598809 administration relative to placebo, in regions including thalamus and dorsal striatum and a decreased BOLD response in areas including insular cortex across all participants. However, contrary to our expectations, no differences were observed between controls and dependent groups in either task-related neural response (as measured under the placebo condition) or in response to D3 antagonism. Instead, we observed subgroup differences in neural response to the task under placebo, such that the polydrug group displayed greater task-related BOLD signal in amygdala and insula/opercular cortex as compared with the alcohol-only group. Similarly, subgroup differences emerged in response to GSK598809 within the D3-rich substantia nigra region: divergent responses were observed with increased and decreased BOLD response to GSK598809 relative to placebo in alcohol-only and polydrug subgroups respectively. In line with expectations, high levels of trait anxiety, perceived stress and childhood adversity were evident in our substance dependent groups relative to controls. Childhood adversity was negatively associated with BOLD response to D3 antagonism, suggesting a modulatory role for this transdiagnostic risk factor for negative affect. Overall findings suggest that the neural response to aversive processing may be differentially expressed in alcohol and alcohol-cocaine polydrug dependence. The tendency for D3 antagonism to increase BOLD response in limbic and D3-rich regions associated with dopaminergic hypofunction could have clinical benefit, but the non-specific effect of D3 antagonism across groups makes it difficult to ascertain whether the response is likely to be restorative in our abstinent population, either in terms of whether it may act to ‘normalise’ brain response, or in its potential for beneficial clinical outcome in substance dependence.

### Negative emotional processing and group differences

We found patterns of BOLD activation in cortical and subcortical areas in response to processing of aversive relative to neutral stimuli in both controls and substance dependent individuals that were in accordance with expectations for a task engaging the neural substrates of negative emotional processing. Thus, a higher BOLD signal was observed in control and substance dependent groups in limbic areas such as the bilateral amygdala, thalamus and hippocampus. Increased BOLD signal was also observed within globus pallidus, left putamen, and right insular cortex. All these regions are associated with emotional processing generally (12), and many with aversive stimuli more specifically (16, 21, 81, 83). By contrast we found decreased BOLD signal in fronto-cortical regions including ACC, mPFC, opercular cortex and frontal pole, i.e., areas associated with regulatory control of emotions. Contrary to expectations, no task-related BOLD response was observed in the NAcc in response to the viewing of aversive > neutral images, despite previous evidence for its involvement in the neural processing of stressful imagery (14). Overall, the task engages the brain ROIs, and findings are consistent with the concept of functional interplay between corticolimbic regions during negative emotional processing.

The emergence of negative affective states and difficulty coping, along with intensified stress- and anxiety- like responses, are important clinical characteristics of substance dependence that serve to initiate and perpetuate drug seeking and relapse, both as a coping mechanism and as a way to avoid the acute negative states associated with withdrawal (84, 85). This “dark side” of addiction remains prominent even in protracted abstinence, with dependent individuals being vulnerable to craving, where drug-, cue-, and stress- reinstatement could lead them to relapse (42). It was therefore surprising that, contrary to our hypothesis, no differences in BOLD signal were observed between control and dependent individuals in response to aversive images, in either the whole brain analysis or in the *a priori* ROIs. The possibility for differences in BOLD response to emotional processing between dependent subgroups relative to controls was anticipated, so a sub-group analysis compared alcohol-only and alcohol-cocaine polydrug groups. These groups are known to differ not only in terms of their lifetime drug use and dependence history, but also in their trait characteristics, with the latter subgroup displaying higher levels of stress vulnerability including childhood adversity and perceived stress relative to controls and alcohol-only. Polydrug dependent individuals more generally suffer from worse prognosis and treatment outcomes; higher rates of relapse, lower treatment retention, higher mortality rates and a greater burden of psychiatric morbidity (86). Thus this distinction may be clinically relevant.

This subgroup comparison revealed increased limbic BOLD response within the polydrug group relative to alcohol-only dependent individuals, which may therefore have masked any difference between the combined dependent group and controls in these regions. Visual exploration of the pattern of BOLD response in subgroups as compared with the control group in the same regions indicated that the difference was driven not only by an increased neural response in polydrug relative to controls but also a lower response in the alcohol-only group relative to controls, although this comparison was not explicitly tested. Subgroup analysis also indicated some divergence in the cortical response between groups, with higher BOLD response within the left insula, pre- and post- central gyrus, opercular cortex and inferior frontal gyrus in polydrug versus alcohol-only subgroups, with a tendency for the latter to be lower as compared with controls. Previous literature in short- and long- term abstinent alcohol dependent individuals supports this finding; studies found lower BOLD response to non-alcohol-related aversive images or negative emotional faces relative to controls (15, 19, 28, 87). Consequently, the lower insula/opercular/postcentral gyral brain activity observed in the alcohol-only group could indicate an attenuation in neural sensitivity to aversive stimuli in this region, which could influence emotion regulation (12).

The heterogeneity of drug dependence in our cohort is a potential explanation for the subgroup differences emerging. The cohort consisted of two distinct dependent populations: alcohol-only and cocaine-alcohol polydrug groups, of which approximately 60% were also opioid dependent. Previous literature has shown different behavioral phenotypes emerge based on patterns and types of drug consumption, so the pattern of brain activity between the two groups might also be expected to vary (9). Stimulant (e.g., cocaine) users have been shown to evaluate negative images as more negative, whereas “sedative” (e.g., alcohol and opioids) users tended to evaluate the same images in a similar way to controls (9). Thus, increased sensitization toward unpleasant images in stimulant users could potentially explain the higher BOLD signal observed in our study of polydrug relative to alcohol-only individuals. Regarding the neural substrates involved in these divergent responses, within the whole brain analysis, the polydrug group presented with higher BOLD response in limbic regions including amygdala, hippocampus, parahippocampal gyrus and left thalamus, all of which have a major role in emotional processing. This increase in BOLD signal could be attributed to cocaine dependence in our polydrug group, given that cocaine dependent individuals have been shown to exhibit higher limbic activity, relative to controls, when experiencing negative emotions (14).

An alternative or perhaps complementary explanation is that transdiagnostic trait characteristics or prodromal risk factors may play a role in the divergent BOLD responses to emotional processing in the polydrug relative to alcohol-only groups. As expected, high levels of perceived stress and childhood adversity were particularly evident in our polydrug cohort (36, 37), and these factors are known to be associated with emotional dysfunction (35), and to influence neural response to emotional stimuli. For example, amygdala hyperreactivity in response to emotional stimuli is commonly described in relation to childhood and adult trauma (31–33), which is consistent with the increased limbic BOLD response observed in the cluster including amygdala/hippocampal/thalamic/dorsal striatum in our polydrug cohort relative to alcohol-only cohorts, the latter of which reported comparable childhood adversity to controls. Indeed, regression analyses revealed a positive association (albeit weak, not surviving more stringent multiple comparison correction) between substantia nigra BOLD response to the task and childhood trauma, suggesting a higher burden of trauma may be associated with enhanced neural responding in this region, which could be reflected in elevated striatal dopamine (88, 89).

The potential emergence of subgroup differences in aversive processing needs to be seen in the context of the lack of effect in the overall group comparison with controls. The absence of alterations in cortical response to aversive processing in particular is not consistent with some previous literature which suggests that reductions in neural response might be expected in both alcohol and cocaine dependent individuals relative to controls (15, 18, 19). Our results are instead more consistent with no overall difference in cortical response between groups as observed by others (22).

### D3 receptor antagonist effect

#### Effect of D3 antagonism on aversive processing in control and substance dependent groups

We expected GSK598809 to normalise brain function by decreasing neural response in areas with higher activity during aversive stimuli and increasing neural response in areas associated with hypofunction. In particular, we expected to observe increased limbic responses and blunted cortical responses in substance dependence that might be ameliorated by D3 antagonism, due to its postulated capacity to restore hypodopaminergic function (38, 51). On the contrary, we saw no such group differences in task-related responding, and no differential response to drug between controls and substance dependence cohorts either at a whole brain level or in *a priori* ROIs. This latter finding was particularly unexpected, given their close association with emotional regulation (amygdala, mPFC, NAcc), and D3 receptor expression (SN, VP, NAcc), both of which were expected to be altered in substance dependence.

Instead, we observed greater BOLD signal across groups in response to GSK598809 in subcortical areas like the bilateral thalamus, caudate, globus pallidus and left putamen. These areas are associated with moderate to high D3 receptor density and are indirectly associated with negative emotional processing (47, 90). The thalamus sends outputs to areas directly associated with negative emotional regulation, including amygdala, mPFC and NAcc (91–93), and acts as a relay center, receiving inputs from the globus pallidus, providing a pivotal role in response inhibition. The caudate is associated with drug craving and habitual learning, which are key components of maintaining substance dependence (84). We also observed BOLD increases in the fusiform, parahippocampal (limbic), and middle temporal gyri, areas with lower D3 receptor expression but that are closely associated with facial processing and recognition, the provision of context during scene visualization, and integration of emotional content respectively. This finding is thus consistent with the viewing of emotive images, many of which include faces. Disruption of response inhibition and emotional regulation and negative affect are key processes thought to underlie substance dependence and may be key drivers of relapse (76), so an enhancement in function in these regions by D3 antagonism could be beneficial. However, the relationship between BOLD response and brain function is far from simple and in the absence of robust evidence of dysregulation in the neural circuitry underpinning emotional processing in this dependent cohort in these specific regions, this interpretation is somewhat speculative.

The observed greater BOLD signal in whole brain analysis was partially reflected in the *a priori* ROI analyses, in which there was a general trend toward higher BOLD response to D3 antagonism in all regions tested. Despite the amygdala being robustly activated during the task and having moderate D3 receptor density (46, 49), no enhanced BOLD effect was seen in this region following GSK598809. Similarly, there were no changes in mPFC or NAcc BOLD response to D3 antagonism. This might seem surprising given the importance of the former in emotional dysregulation, and the high D3 receptor expression levels of the latter (46). It is possible that insufficient receptor numbers are present for the drug to alter brain activity in PFC; D3 receptors are relatively sparse, and dopamine may be less tightly regulated in this area (39), both of which might impact on GSK598809’s ability to exert an effect in this region. Perhaps more surprising is the lack of NAcc BOLD signal change following D3 antagonism. However, since no task-related BOLD response was observed in the NAcc in response to the viewing of aversive > neutral images, modulation of response by pharmacological manipulation might not be anticipated here either. In addition, it is possible that dopaminergic function within NAcc is relatively unaffected by D3 receptor antagonism locally, and is less likely to be influenced by changes in mesolimbic innervation since D3 receptors are largely absent from the ventral tegmental area (94). The absence of D3 receptors within VTA neurones is also in line with the lack of effect in the afferent PFC regions to which they project.

It is important to note that we have previously reported increased BOLD response following GSK598809 administration in the same cohort in response to monetary reward anticipation (51). In line with our results, this effect of drug to ‘restore’ function was seen in both substance dependent individuals and controls (overall effect of drug but no drug*group interaction in whole brain analysis). The fact that in both cases, i.e., during reward anticipation and negative emotional processing, an increase in neural response was observed following D3 antagonism, points to the possibility of a common D3-related effect on BOLD response across tasks. This could involve mechanistic similarities, e.g., a common effect of D3 antagonism to increase blood flow in areas of high receptor density, and/or the capacity for D3 antagonism to affect BOLD response more generally. Considering that the areas showing increased BOLD activity during aversive stimuli presentation were not all associated with negative emotional processing, we might speculate that this increased activity could be attributed to a general dopaminergic effect. Knowing that D3 receptors act as autoreceptors, inhibiting DA synthesis and release (95), their inhibition by GSK598809 would be expected to lead to an increase in extracellular dopamine and thus conceivably leading to an increased neural response, consistent with our results and those of Murphy et al. (51). Drugs that increase extracellular dopamine, such as D3 antagonists, have been shown to improve response inhibition in alcohol and cocaine dependence (96), suggestive of a promising therapeutic approach (38, 51). We therefore speculate that GSK598809 has a restorative function, but instead of decreasing the BOLD signal in areas with high activity, it acts to restore the hypodopaminergic state seen in addiction (25) which manifests as an increase in neural response.

Interestingly, lower BOLD signal change was observed in the opercular and insular cortices in response to GSK598809 in our cohort. These areas have previously been shown to be associated with negative emotional processing in controls, cocaine and alcohol dependent individuals (18, 19, 22), but not with high D3 receptor levels. Increased activity in the insula is associated with drug-craving (97), and a recent study conducted in alcohol dependent rats showed that D3 antagonism attenuated abnormal insular cortex resting-state functional connectivity (98). Information about craving was not collected in this acute dosing study, but decreased insular cortex activity in response to selective D3 antagonism could explain the partial alleviation of nicotine craving in short-term abstinent smokers (70).

#### Effect of D3 antagonism on aversive processing in alcohol-only versus polydrug subgroups

Whilst there were no significant effects of D3 antagonism when comparing controls with the substance dependent group as a whole, differences emerged when comparing the response to drug between the alcohol-only versus polydrug subgroups. Divergent responses emerged in the polydrug dependent group which demonstrated no change in BOLD signal following GSK598809, in contrast to the alcohol-only and control groups where greater BOLD response was observed across ROIs in response to D3 antagonism. The effect also varied according to ROI, and was particularly evident in D3-rich areas, notably substantia nigra.

Increased BOLD response was observed bilaterally within SN in response to GSK598809 in the alcohol-only group, in line with that of controls. SN is the area of highest D3 receptor density in the human brain (46), and is associated with alterations in substance dependence. Indeed, the evidence for D3 receptor alterations in substantia nigra is more robust in cocaine/stimulant dependence than in alcoholism. Increased D3 receptor availability has been found in cocaine dependent post mortem brains (56) and in PET studies of cocaine dependence (52, 54), consistent with preclinical evidence of upregulation in D3 receptors following exposure to stimulants in rodents (99). Whereas there is no evidence of upregulation of substantia nigral D3 receptors in alcohol dependence (47, 100). Thus, the presence of a greater number of D3 receptors in this region could influence the neural response to emotional processing following selective antagonism at this receptor, and points to possible abnormal expression or function of D3 receptors in the polydrug dependent cohort.

#### Relationship between neural responses, anxiety and childhood trauma

Our data suggest that certain specific clinical trait differences may play a role in the apparent difference in response to D3 antagonism in polydrug versus alcohol-only subgroups from our ROI analysis. Anxiety is highly comorbid in substance dependence, and is thought to be a partial mediator of the disorder (32, 76, 101), and both of our substance dependent populations, had significantly higher trait anxiety scores compared to controls. As was the case for childhood adversity, there is a suggestion that baseline trait anxiety may moderate the response to D3 antagonism in substantia nigra and ventral pallidum. The correlation between these measures and response to drug in D3-rich regions of the SN and VP suggests that mental health traits could have a modulatory effect on D3-receptor related emotional processing that may be independent of substance dependence *per se*. Associations are weak, and only the negative D3-SN association survived the more astringent correction for multiple comparisons, so the findings should be treated with caution, but could be interpreted as a sign that those with increased burden of negative affect may respond less to D3 antagonism, rather than deriving greater benefit, as one would hope for a medication targeting negative affect in substance dependence. An alternative explanation could be that the moderation of the effect is instead indicative of underlying differences in D3 receptor expression; the divergent effects of D3 antagonism by subgroup, occurred in the two areas of highest D3 receptor density in human brain (SN and VP), and in areas where alterations in D3 receptor availability are robustly described in cocaine dependence, as previously described. This could point to a previously unrecognised relationship between D3 receptor function and aversive processing. The possibility that experience of childhood adversity may negatively impact on neural response to GSK598809 has implications for treatment utility in addressing the specific problem of negative affect in addiction, as this is one of the core drivers of continuity and a risk factor for relapse.

### Further considerations and limitations

It is vitally important to study real-world substance dependent populations that would most benefit from new interventions and that have the greatest unmet need. However, there are inherent limitations in studying complex cohorts. The heterogeneity of the recruited sample can be viewed as a strength of this study because they are representative of this target population in the UK, however it also presents challenges in the interpretation of findings, and so patient selection should be considered carefully in future studies. This study focused on considering the potential for a differential impact of alcohol and cocaine dependence on the neural processing of emotion and response to D3 antagonism, however, it is important to view these dependent cohorts as part of the wider clinical picture of heterogeneity. For example, it should be noted that more than 50% of the polydrug group were also opioid dependent. Exposure to heroin has been shown to decrease the expression of D3 receptor mRNA and protein levels in the caudate, putamen and NAcc in mice (102). If this were to translate into lower D3 receptor density in cocaine dependence, this might be expected to impact on the actions of D3 selective antagonists on neural processing in our polydrug dependent sample. Similarly, almost all polydrug dependent individuals are, or have been dependent smokers; this is also true for the vast majority of alcohol-only dependent individuals and is applicable in this cohort but also more broadly. Whilst a great deal of effort was placed into including a comparable number of smokers in the control group, lifetime exposure to nicotine was demonstrably higher in the dependent (particularly polydrug) group, which could also impact our findings since nicotine dependence may influence emotional processing, e.g., evidence of alterations in amygdala functional connectivity (103). Years of cumulative exposure to substances also contribute to differential volumetric changes in important frontal areas like the PFC, ACC, and insula (104, 105), although the nature and extent of the assumed impact on function within these regions is less well understood. Length of abstinence may be an important factor when comparing this study with the wider literature; many study short-term abstinence with the view that these individuals are more vulnerable to relapse e.g., (22), thus are not directly comparable with our cohort. Whilst instability is undoubtedly highest in early abstinence, relapse rates are still stubbornly high even after protracted abstinence; 12 of the 36 dependent individuals taking part in this study are known to have relapsed within one year after taking part (data not shown), and some studies suggest that 5 years of abstinence is required before stable recovery can be achieved (106). The fact that the individuals in our cohort were relatively long-term abstinent (median 9 years, range 1–102 months) is important to note, because it suggests that emotional dysregulation may persist into abstinence, with enduring impact on the potential for recovery and risk of relapse.

Between group and drug differences could be explained by other factors. For example, despite the exclusion of active mental health disorders from both control and dependent groups, the burden of lifetime mental health difficulties in the dependent group is significantly greater than that of controls. Such clinical factors may influence or confound neural differences observed between groups.

Our findings should be interpreted in light of several limitations. Sample size is one consideration; whilst the sample was comparable with similar studies, a larger sample would have provided more robust evidence, especially given the divergent effects observed across sub-groups (*n* = 17 polydrug and *n* = 19 alcohol-only). Secondly, the pseudorandomised cross-over design also has the potential to introduce an order effect; the D3 antagonist was always administered at a later visit (session 4 or 5), as compared with placebo (session 2 or 3). The design was deliberately biased in this way to avoid the potential for lost placebo data due to low retention rates (to conserve the ability for comparison with the other drugs under study; GSK598809, naltrexone and aprepitant/vofopitant). Ultimately retention rates were higher than anticipated, so data loss was not an issue (76). Habituation was minimised through the use of novel images at each session, and the randomisation of images presented at each visit (the same images were not shown at every placebo session, or at every D3 session). The fact that both increases and decreases in BOLD response were observed in response to drug, that not all ROIs responded to drug in the same direction, and that a divergence of response occurred across groups makes the possibility of a global order effect seem unlikely. Finally, since 60 mg GSK598809 only partially blocks the total available brain D3 receptors (70), it is probable that the dose used in our study was insufficient to occupy all available D3 receptors, such that a higher dose could prove more beneficial in future research.

## Conclusion

Addressing the specific problem of negative affect in addiction is important, as it is one of the core drivers of continuity, is a significant risk factor in relapse and with few therapeutic tools available for treatment. Our results suggest that the D3 antagonist GSK598809 enhances limbic and D3-related BOLD response during negative emotional processing in controls and long-term abstinent substance dependent individuals, which may be primarily driven by alcohol-only rather than polydrug dependent individuals. This demonstrates that GSK598809 modulates relevant brain circuitry and is consistent with the interpretation that D3 antagonism may enhance dopaminergic function and therefore may have clinical therapeutic potential. However, the lack of group differences in response to negative emotional processing and the non-selective effects of D3 antagonism across groups was somewhat disappointing, as it was hoped that a lack of effect in controls, coupled with an attenuation of dysregulated neural response in substance dependence might guide interpretation as to the sensitivity and specificity of the D3 antagonist effect, and be suggestive of its efficacy in normalising aberrant emotional responding. This would have helped to determine whether any modulation in emotional processing by D3 antagonism was likely to be clinically beneficial and provided direct support for its further validation as a target for treatment of negative affective states in substance dependence. With these findings alone, it is not possible to ascertain whether the effect of D3 antagonism is likely to be beneficial or not, so further research is warranted. Further, the potential for divergent neural responses within the dependent cohort according to drug(s) of dependence, and the possible impact of transdiagnostic trait characteristics on the response to drug is deserving of further exploration.

## Data availability statement

Anonymised data that support the findings of this study are available on request from the corresponding author. The raw imaging and individual-level data are not publicly available due to privacy or ethical restrictions. Requests to access the datasets should be directed to LP, l.paterson@imperial.ac.uk.

## Ethics statement

The studies involving human participants were reviewed and approved by West London and Gene Therapy Advisory Committee National Research Ethics Service Committee (REC reference: 11/H0707/9). All participants provided their written informed consent to participate in this study.

## Author contributions

IV and LP wrote the manuscript. LP, LF, and IV conceived of the presented idea, analytical approach, and hypotheses tested. LF, IV, AH, and LP performed the analyses. LP supervised the analysis. AM provided the ROIs. LP, JM, RF, CO, AM, DS, and ET were involved in data collection. RE, KE, JS, TR, BD, AL-H, and DN conceived of the overall ICCAM project and designed the over-arching study. TR, BD, DN, and AL-H obtained the funding. All authors approved the manuscript.

## Footnotes

1
https://csea.phhp.ufl.edu/media/iapsmessage.html
