# Supplementary material for: Selective D3 receptor antagonism modulates neural response during negative emotional processing in substance dependence
Source: Front Psychiatry. 2022 Oct 19;13:998844. doi: 10.3389/fpsyt.2022.998844 (PMC9627287; doi:10.3389/fpsyt.2022.998844)
Supplement: Supplementary file 1 [file Data_Sheet_1.docx]

Supplementary material

Regions of interest

*A priori* regions of interest (ROIs) were selected according to their association with negative emotional processing and/or high D3 receptor expression, and based on our previous publication in which D3 antagonism with GSK598809 was shown to modulate monetary reward processing (Murphy et al., 2017). The bilateral amygdala, bilateral NAcc and mPFC were chosen due to their association with aversive and negative emotional processing, and the pallidum and substantia nigra due to their high D3 receptor expression levels according to PET studies (Tziortzi et al., 2011; Murphy et al., 2017).

For the bilateral accumbens, mPFC and amygdala, anatomical ROIs were defined using the Harvard-Oxford cortical and subcortical structural atlas. As the cortical atlas does not contain the mPFC, masks of the frontal medial cortex and anterior cingulate cortex (ACC) were created. Using fslmaths, the ACC was subtracted from the frontal medial cortex to create the mPFC mask. Masks were then thresholded at 50% and binarised. The ventral pallidum and ventral striatal ROIs were defined according to Murphy et al., 2017, based on PET work by Tziortzi et al, 2011. They were hand drawn onto the Ch2better template within MRIcron (Rorden, [www.mricron.com](http://www.mricron.com)). The substantia nigra mask was taken from the Hammersmith probabilistic atlas© Copyright Imperial College of Science, Technology and Medicine 2007, (Hammers et al 2008). Available from [www.brain-development.org](http://www.brain-development.org). Masks are shown in Figure S1.


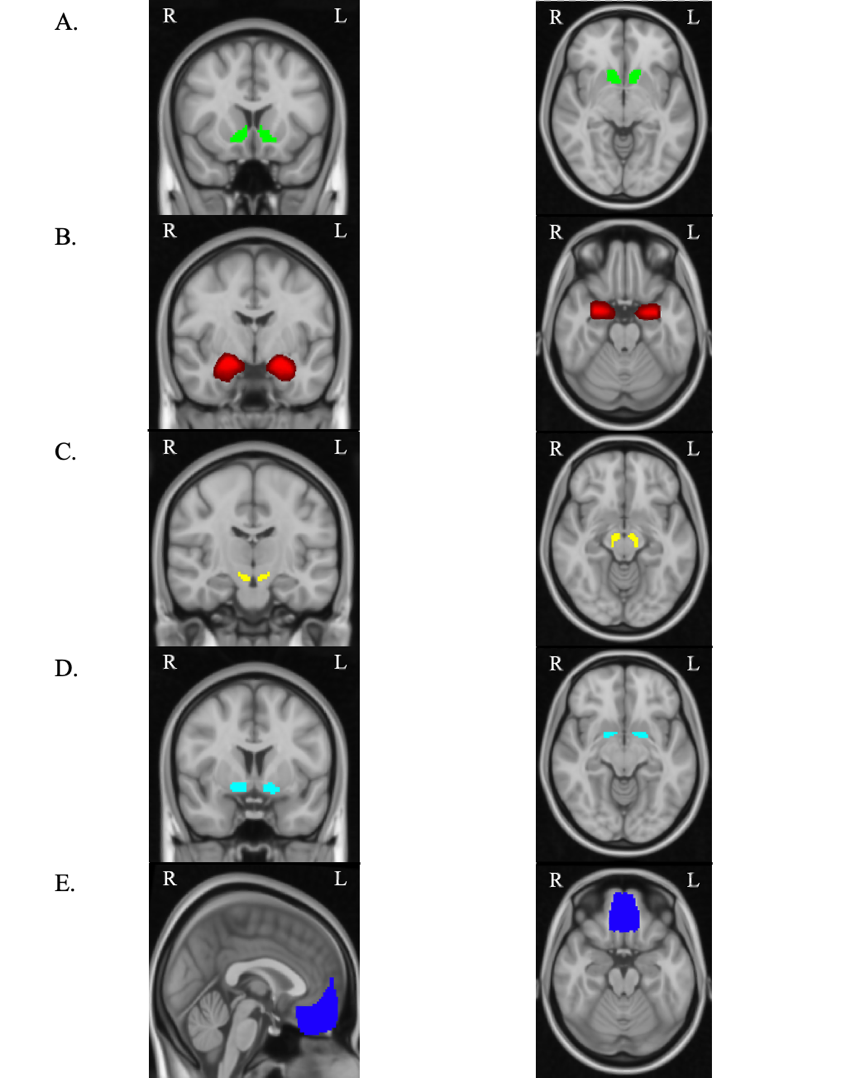


**Figure S1.** Anatomical ROIs.

(A) Ventral striatum

MNI coordinates: x=-0.1, y=14.0, z=-5.6.

(B) Amygdala

MNI coordinates: x=0.5, y=-5.3, z=-21.8.

(C) Substantia nigra

MNI coordinates: x=5.2, y=-16.1, z=-12.4,

(D) Ventral pallidum

MNI coordinates: x=-18.6, y=4.1, z=-9.6,

(E) Medial prefrontal cortex

MNI coordinates: x=-0.05, y=40.6, z=-19.6.


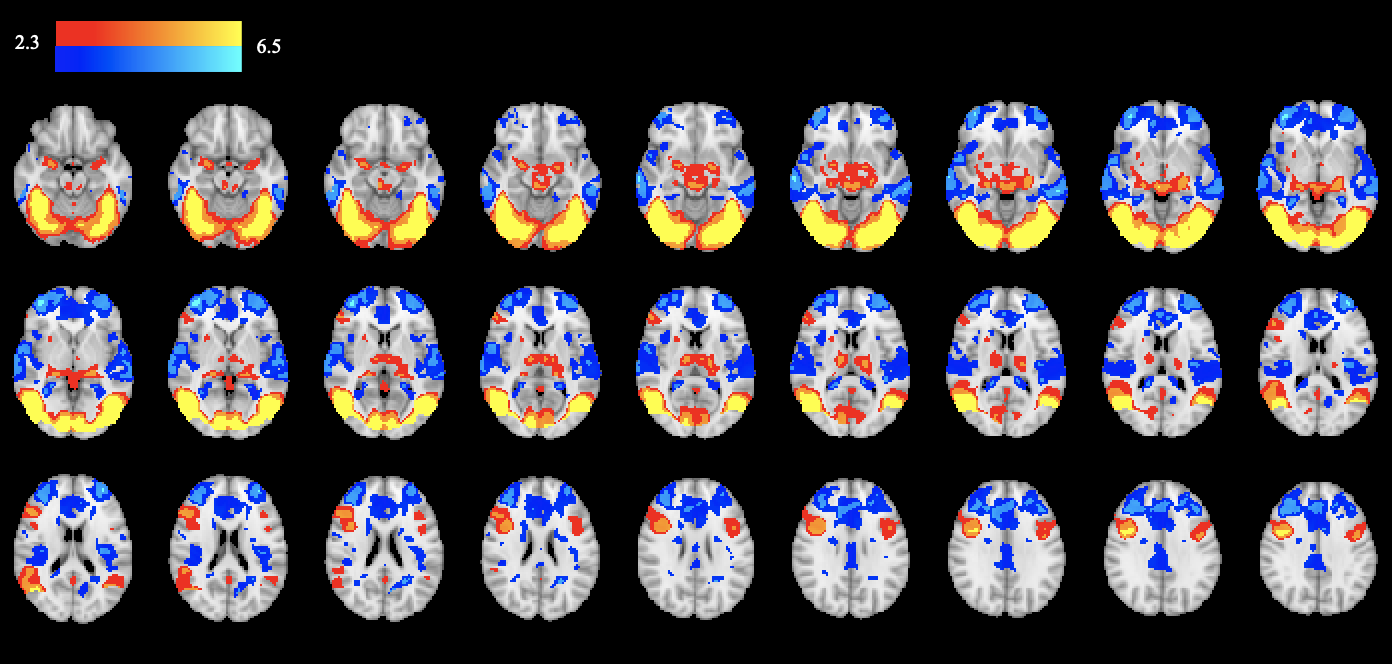


**Figure S2.** Whole brain activation maps for average BOLD response in aversive>neutral contrast in HC and DD groups (n=68). Red/yellow colours represent increased BOLD response and blue colours indicate decreased BOLD response. Z>2.3, P<0.05 corrected.

**Table S1. Cluster maxima co-ordinates for average BOLD signal in the EIT aversive > neutral contrast under placebo across all individuals**

|  | **Cluster No.** | **Location of peak voxel** | **#Voxels** | **P** | **Weighted centre MNI coordinates (mm)** | | |
| --- | --- | --- | --- | --- | --- | --- | --- |
|  |  |  |  |  | **X** | **Y** | **Z** |
| (A) | 3 | Right lateral occipital cortex | 25266 | 1.34E-33 | 46 | -74 | -4 |
|  | 2 | Right precentral gyrus | 2320 | 3.58E-06 | 46 | 4 | 34 |
|  | 1 | Left precentral gyrus | 973 | 0.00428 | -46 | 0 | 36 |
| (B) | 6 | Right frontal pole | 15101 | 1.34E-23 | 38 | 54 | 0 |
|  | 5 | Left middle temporal gyrus | 5030 | 6.60E-11 | -60 | -34 | -6 |
|  | 4 | Right middle temporal gyrus | 4515 | 4.32E-10 | 66 | -20 | -10 |
|  | 3 | Right cerebellum | 824 | 0.011 | 40 | -60 | -40 |
|  | 2 | Left cerebellum | 694 | 0.0262 | -44 | -66 | -36 |
|  | 1 | Right cingulate gyrus | 616 | 0.0451 | 4 | -30 | 34 |

(A) Clusters show where there is an overall higher BOLD signal in the average group maps across HC and DD groups. (B) Clusters show where there is an overall lower BOLD signal in the average group maps across HC and DD groups. Table shows cluster number, location of peak voxel according to Harvard-Oxford atlas, number of voxels within the cluster, P value and MNI co-ordinates for peak voxel.

**Table S2. No significant effect of GSK598809 in ROIs between HC and DD groups.**

A)

| ROI | Healthy controls (n=32) | | Drug Dependent (n=36) | |
| --- | --- | --- | --- | --- |
|  | Placebo | D3 | Placebo | D3 |
| Amygdala | 0.0477±0.188 | 0.0483±0.312 | 0.0721±0.232 | 0.126±0.210 |
| Ventral striatum (NAcc) | -0.0559±0.188 | -0.0859±0.188 | 0.0011±0.154 | -0.0250±0.184 |
| R ventral pallidum | 0.0498±0.237 | -4E-6±0.298 | 0.0076±0.239 | 0.0872±0.273 |
| L ventral pallidum | -0.0009±0.231 | 0.0375±0.328 | 0.0589±0.260 | 0.166±0.328 |
| R substantia nigra | 0.0411±0.189 | 0.162±0.293 | 0.0613±0.243 | 0.109±0.280 |
| L substantia nigra | 0.0537±0.209 | 0.116±0.244 | 0.0569±0.205 | 0.0839±0.243 |
| mPFC | 0.0310±0.273 | 0.122±0.353 | 0.060±0.241 | 0.112±0.295 |

B)

| Multivariate Tests; HC vs DD | |  |  |  |  |  |
| --- | --- | --- | --- | --- | --- | --- |
| Effect |  | Value | F | Hypothesis df | Error df | Sig. |
| drug | Pillai's Trace | 0 | .010b | 1 | 63 | 0.92 |
| drug * Group | Pillai's Trace | 0.001 | .035b | 1 | 63 | 0.852 |
| ROI | Pillai's Trace | 0.063 | .654b | 6 | 58 | 0.687 |
| ROI * Group | Pillai's Trace | 0.147 | 1.659b | 6 | 58 | 0.147 |
| drug * ROI | Pillai's Trace | 0.079 | .829b | 6 | 58 | 0.553 |
| drug * ROI * Group | Pillai's Trace | 0.157 | 1.794b | 6 | 58 | 0.116 |

A) Mean (SD) % BOLD signal change in ROIs in HC vs DD groups. B) Pillai’s trace results of multivariate analysis showing no effect of drug, group or ROI or any interactions in HC vs DD groups, whilst controlling for centre and age. Design: Intercept + Centre1 + Centre2 + Age + Group. Within Subjects Design: drug + ROI + drug*ROI.

**Table S3. Cluster maxima coordinates for average BOLD signal in the EIT aversive > neutral contrast under placebo.**

| **Cluster No.** | **Location of Peak Voxel** | **#Voxels** | **P** | **Weighted centre MNI coordinates (mm)** | | |
| --- | --- | --- | --- | --- | --- | --- |
|  |  |  |  | **X** | **Y** | **Z** |
| 2 | Brainstem | 3665 | 7.23E-09 | 12 | -14 | -16 |
| 1 | Central opercular cortex | 630 | 0.0371 | -32 | 10 | 16 |

Clusters show where there is an overall higher BOLD signal in the PD relative to the AO group.

**Table S4. Significant effect of GSK598809 in substantia nigra between PD and AO groups.**

A)

| ROI | Polydrug (PD, n=17) | | Alcohol-only (AO, n=19) | | Group comparisons |
| --- | --- | --- | --- | --- | --- |
|  | Placebo | D3 | Placebo | D3 | ^a^Drug, ^b^Interaction, ^c^Group effects |
| Amygdala | 0.142±0.214 | 0.136±0.245 | 0.0095±0.234 | 0.117±0.178 | F_(1,31)_=3.227, p=0.082^a^  F_(1,31)_=2.350, p=0.135^b^  F_(1,31)_=0.733, p=0.398^c^ |
| Ventral striatum (NAcc) | -0.0014±0.169 | -0.0691±0.232 | 0.0033±0.143 | 0.0145±0.121 | F_(1,31)_=0.078, p=0.782^a^  F_(1,31)_=1.981, p=0.169^b^  F_(1,31)_=1.499, p=0.230^c^ |
| R ventral pallidum | 0.064±0.249 | 0.0594±0.336 | -0.0429±0.224 | 0.112±0.207 | F_(1,31)_=0.011, p=0.918^a^  F_(1,31)_=2.195, p=0.149^b^  F_(1,31)_=0.185, p=0.670^c^ |
| L ventral pallidum | 0.112±0.271 | 0.197±0.398 | 0.0117±0.248 | 0.139±0.259 | F_(1,31)_=1.221, p=0.278^a^  F_(1,31)_=0.348, p=0.560^b^  F_(1,31)_=0.538, p=0.469^c^ |
| R substantia nigra | 0.207±0.231 | 0.114±0.352 | -0.0692±0.173 | 0.105±0.207 | **F_(1,31)_=8.327, p=0.007^a^**  **F_(1,31)_=17.515, p<0.001^b^**  F_(1,31)_=3.321, p=0.078^c^ |
| L sub. nigra | 0.141±0.229 | 0.0775±0.301 | -0.0187±0.148 | 0.0897±0.185 | F_(1,31)_=2.434, p=0.129^a^  **F_(1,31)_=6.365, p=0.017^b^**  F_(1,31)_=0.901, p=0.350^c^ |
| mPFC | 0.109±0.274 | 0.0882±0.339 | 0.0171±0.204 | 0.134±0.258 | F_(1,31)_=0.296, p=0.590^a^  F_(1,31)_=1.278, p=0.267^b^  F_(1,31)_=0.150, p=0.701^c^ |

B)

| Multivariate Tests; AO vs PD | |  |  |  |  |  |
| --- | --- | --- | --- | --- | --- | --- |
| Effect |  | Value | F | Hypothesis df | Error df | Sig. |
| drug | Pillai's Trace | 0.083 | 2.805b | 1 | 31 | 0.104 |
| **drug * Subgroup** | **Pillai's Trace** | **0.175** | **6.591b** | **1** | **31** | **0.015** |
| ROI | Pillai's Trace | 0.253 | 1.467b | 6 | 26 | 0.228 |
| ROI * Subgroup | Pillai's Trace | 0.342 | 2.250b | 6 | 26 | 0.07 |
| **drug * ROI** | **Pillai's Trace** | **0.384** | **2.696b** | **6** | **26** | **0.036** |
| **drug * ROI * Subgroup** | **Pillai's Trace** | **0.38** | **2.659b** | **6** | **26** | **0.038** |

Tests of Between-subjects effects:

| Source | Type III Sum of Squares | df | Mean Square | F | Sig. |
| --- | --- | --- | --- | --- | --- |
| Subgroup | 0.21 | 1 | 0.21 | 0.73 | 0.399 |

A) Mean (SD) % BOLD signal change in ROIs in PD vs AO groups.

B) Results of multivariate analysis (Pillai’s trace) showing outcomes for effect of drug, group, ROI and interactions in PD vs AO groups across all ROIs, whilst controlling for centre and age. Design: Intercept + Centre1 + Centre2 + Age + Group. Within Subjects Design: drug + ROI + drug*ROI. Given the overall significant effect of drug*subgroup, drug*ROI and drug*ROI*subgroup (shown in bold type), to explore the interactions, data were further analysed using a mixed 2x2 ANOVA within each ROI with drug as repeated measure and group as between subjects measure, controlling for centre and age. Outcomes of the 2x2 mixed ANOVA are shown in A, and significant effects denoted in bold type.

**Table S5. Cluster maxima coordinates for EIT aversive > neutral contrast for the effect of D3 receptor antagonist GSK598809 relative to placebo.**

|  | **Cluster No.** | **Regions** | **Voxels** | **P** | **Weighted centre MNI coordinates (mm)** | | |
| --- | --- | --- | --- | --- | --- | --- | --- |
|  |  |  |  |  | **X** | **Y** | **Z** |
| (A) | 3 | Lingual gyrus | 4930 | 1.07E-10 | 4 | -86 | -4 |
|  | 2 | Right thalamus | 715 | 0.0227 | 4 | -6 | 0 |
|  | 1 | Right middle  temporal gyrus * | 649 | 0.0357 | 46 | -20 | -16 |
| (B) | 1 | Parietal opercular cortex | 723 | 0.0215 | 38 | -36 | 20 |

A) Peak co-ordinates representing 3 clusters with an overall increase in BOLD signal across HC and DD groups, and B) single cluster where there was a decrease in BOLD signal in response to the drug.***** The cluster peak (MNI coordinates represented) falls in the white matter

1. B)


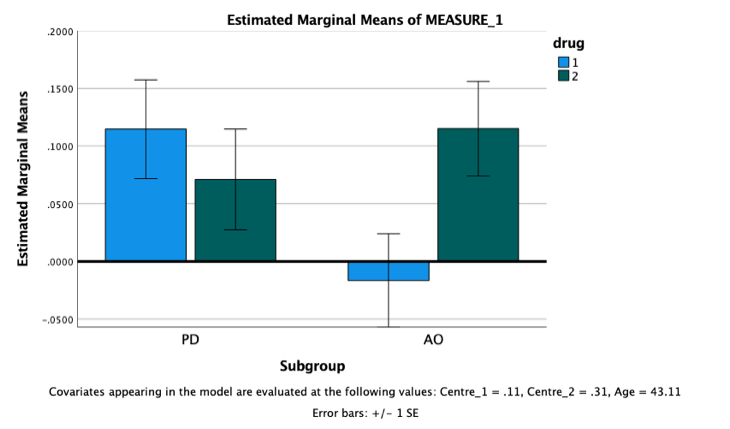

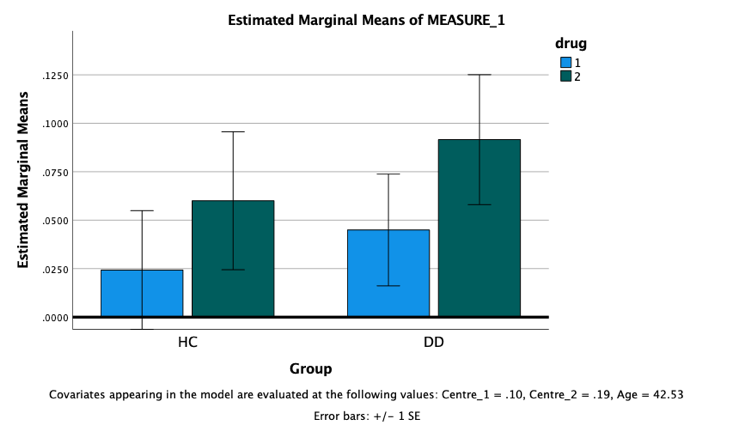


**Figure S2. Overall effect of drug on BOLD response across ROIs differs according to subgroup**. Results from multivariate repeated measures analysis with drug and ROI as within-subjects factors, and group or subgroup as between-subjects factor, controlling for scan centre and age. A) In comparing the effect of D3 antagonism on HC and DD groups (drug 1=placebo, drug2=GSK598809), there were no significant drug, group, ROI or interaction effects. However, the same analysis in B) revealed a differential effect of D3 antagonist on ROI BOLD signal in AO vs PD subgroups; a significant drug*subgroup interaction (p=0.015) was observed with an increase in BOLD response to drug evident in AO relative to PD groups suggesting that the effect of GSK598809 to increase BOLD response in these ROIs is driven primarily by the AO group.


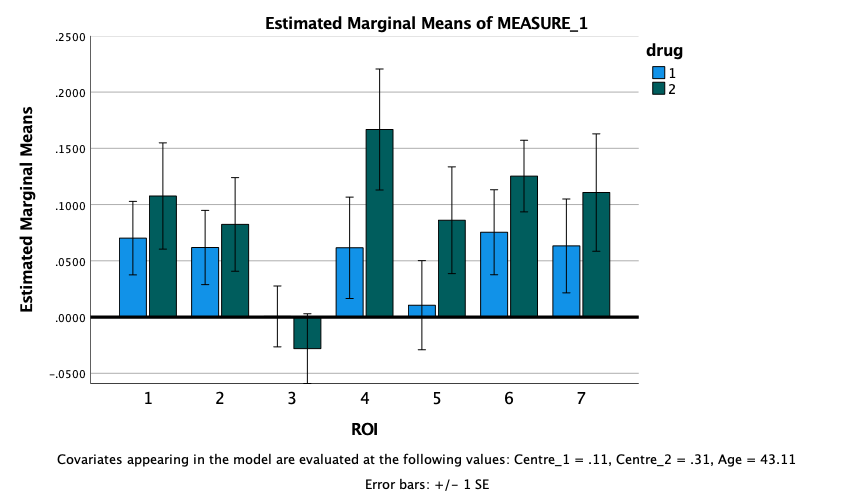


**Figure S3; Differential effect of drug by ROI across groups.** A significant drug*ROI interaction (p=0.036) was observed. Results show increased response to drug in all ROIs except the ventral striatum in which there is a no change/reduction. However, a subgroup*drug*ROI interaction was also observed suggesting differential effect of drug according to ROI and subgroup, requiring further exploration. Drug 1=placebo, drug 2=GSK598809, ROIs as follows: 1) Right_SN, 2) Left_SN, 3) VS, 4) Left_VP, 5) Right_VP, 6) Amygdala, 7) mPFC.

**Figure S4 Comparison of effect of D3 antagonism by ROI in AO vs PD subgroups with controls.** This figure is identical to Figure 4 in the main paper but also includes the control group for visual reference/comparison. No significance testing for comparison with controls was performed. Data are % BOLD signal change during the aversive > neutral contrast following administration of placebo and GSK598809 across PD (n=17), AO (n=19) and HC (n=32) groups. Values are estimated marginal means (± SEM).

**Table S6A. Associations between BOLD response to evocative task (under placebo) and CTQ scores**


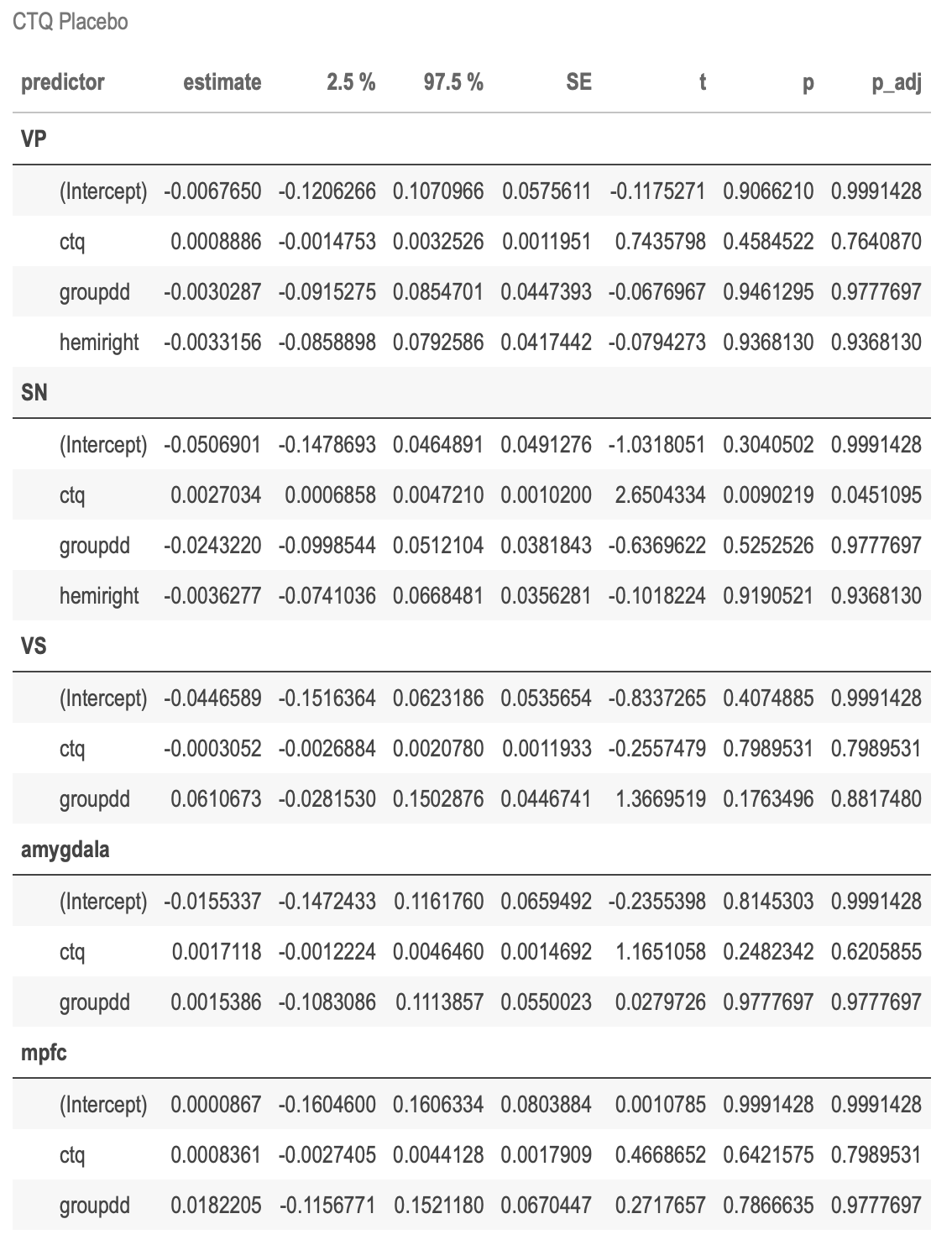


Tabulated results in tables 6 and 7 are outcomes from linear regression models controlling for group and hemisphere (as appropriate). Data presented are predictor (dependent variables included in the model), estimate (B estimate for effect size), 2.5 and 97.5% confidence intervals for B estimate, standard error, t value, uncorrected p value and adjusted p value (FDR corrected for number of tests performed).

**Table S6B. Associations between BOLD response to evocative task (under placebo) and STAI-T scores**


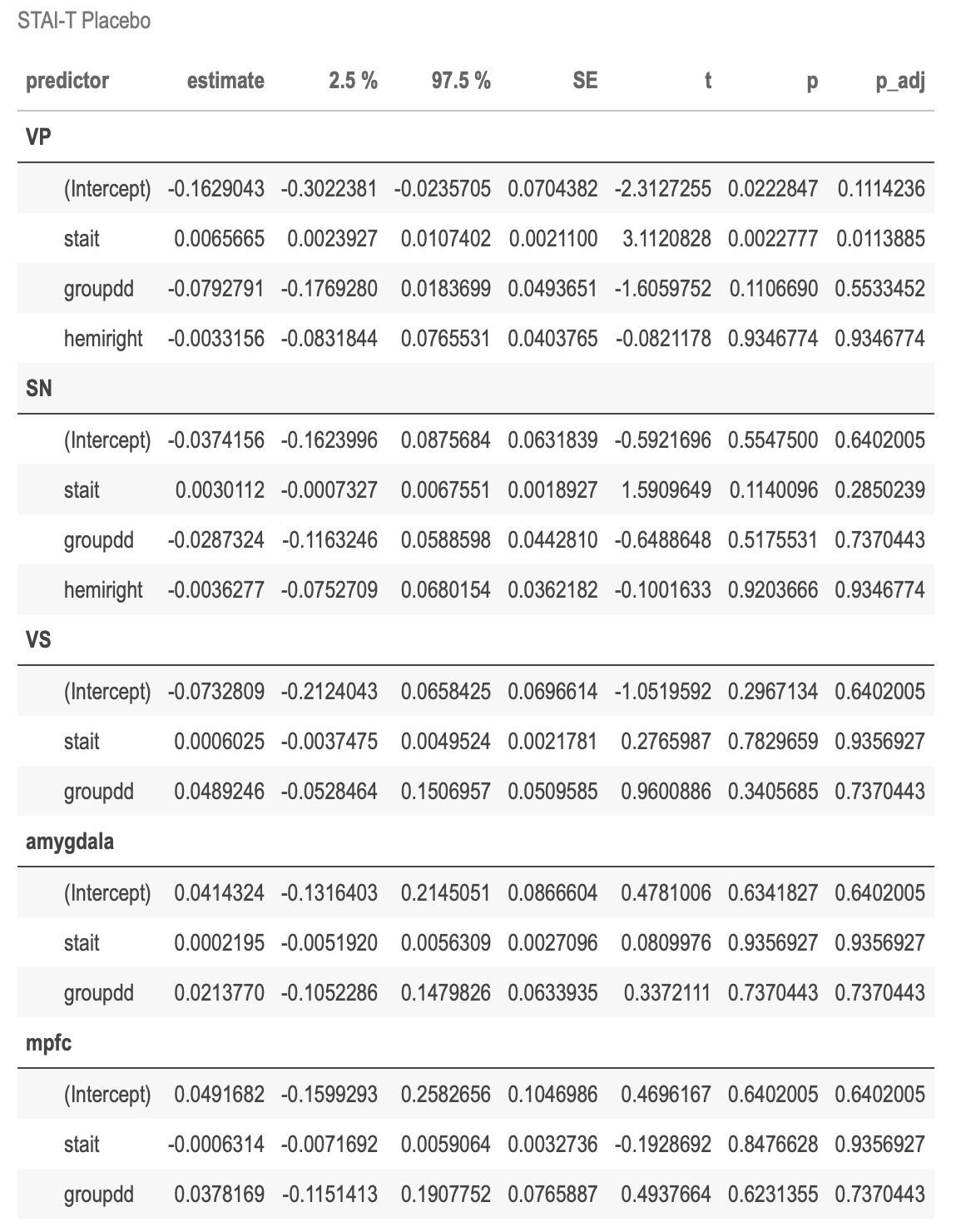


**Table S7A. Associations between BOLD response to D3 antagonism during the evocative task (drug-placebo) and CTQ scores**


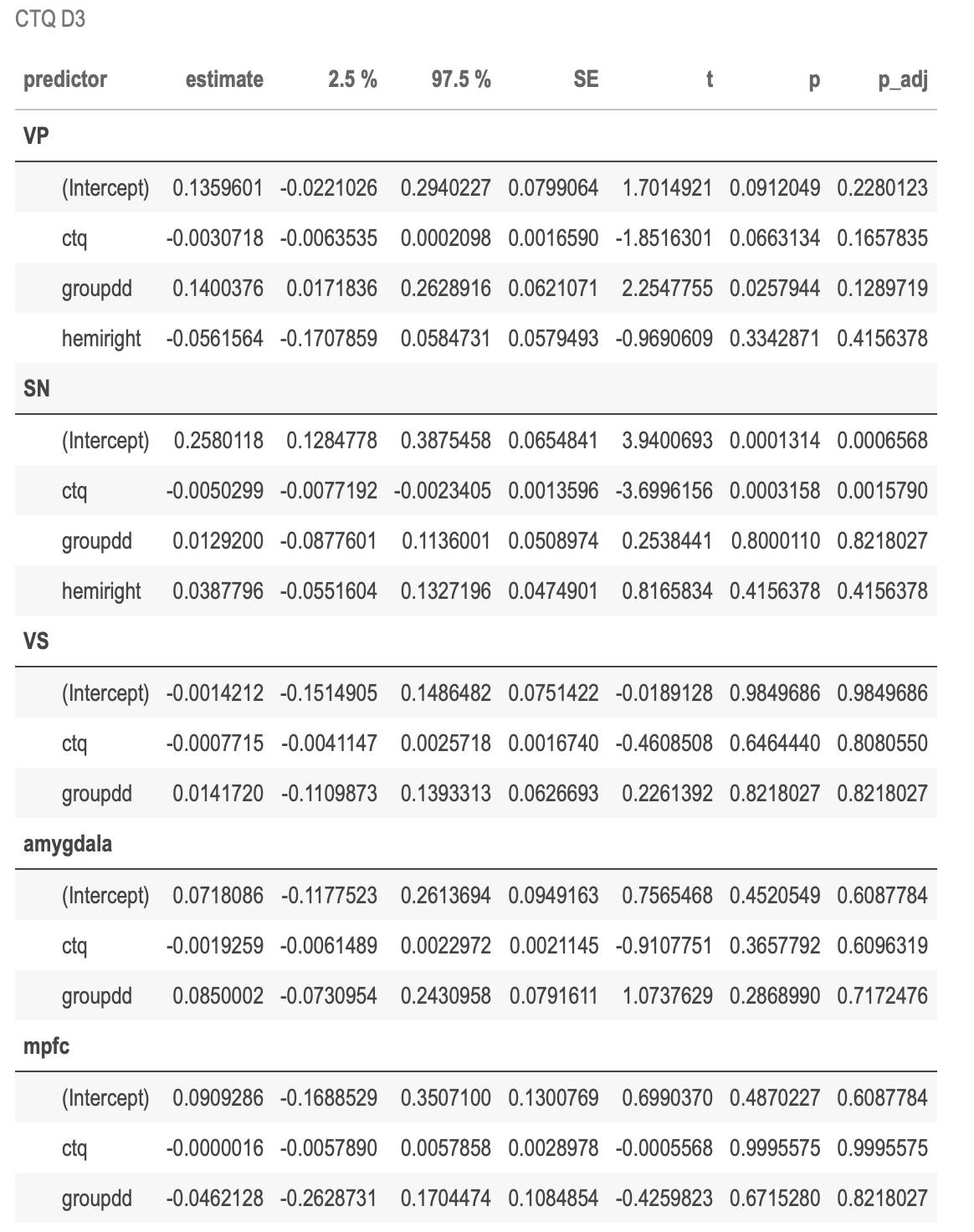


**Table S7B. Associations between BOLD response to D3 antagonism during the evocative task (drug-placebo) and STAI-T scores**


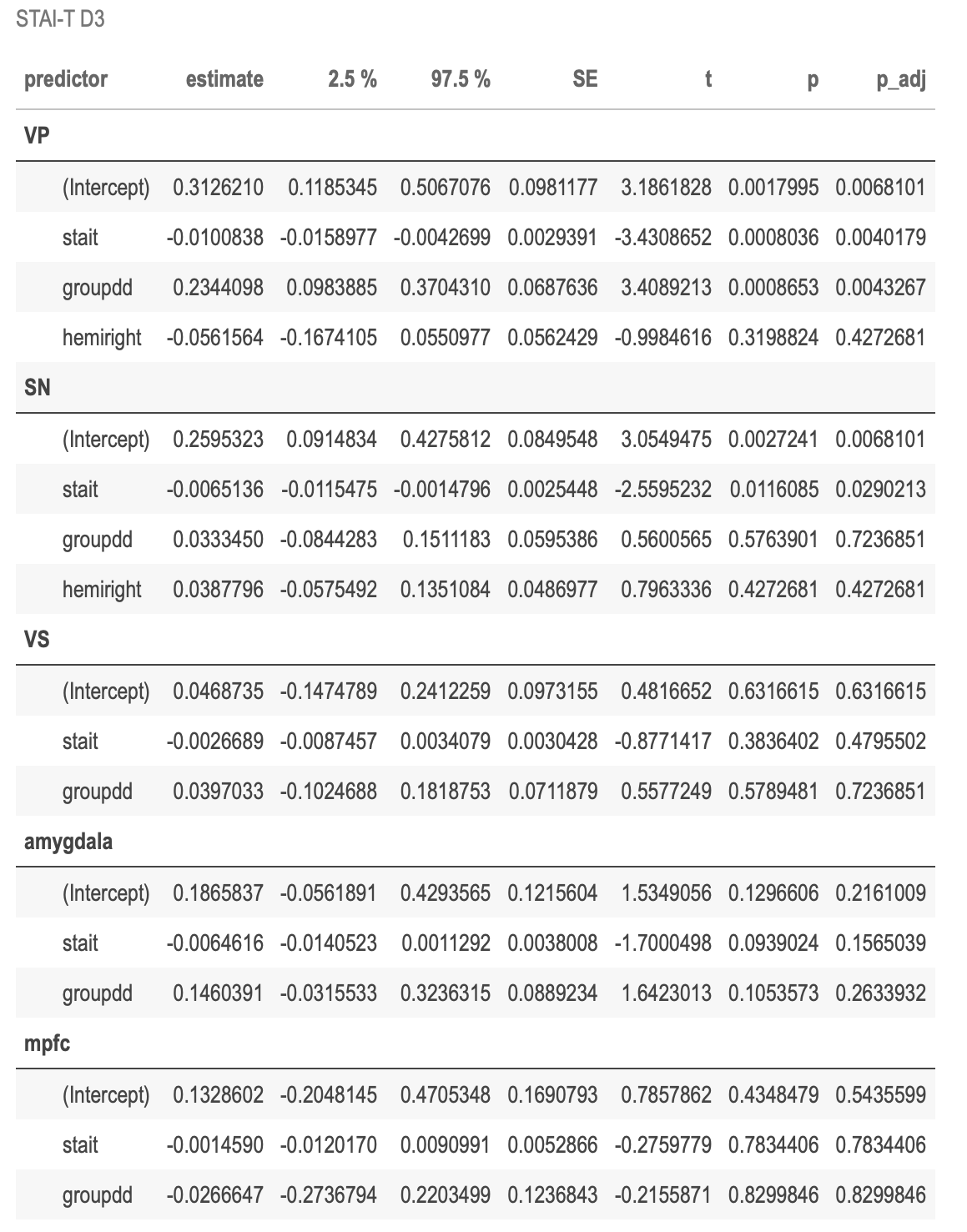


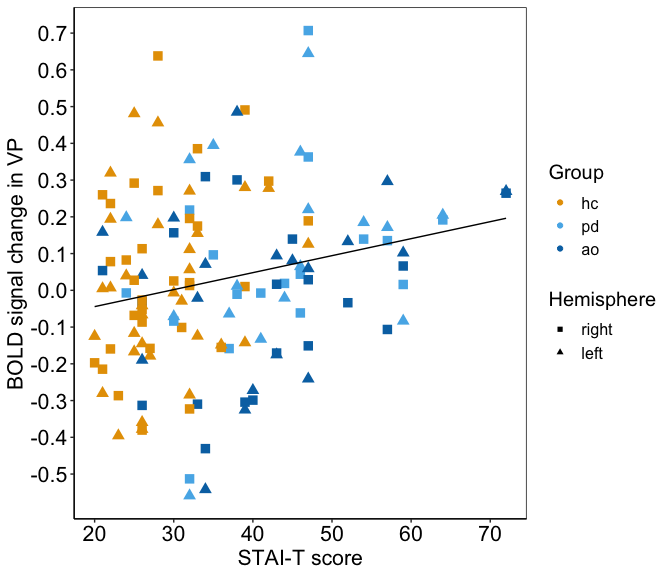

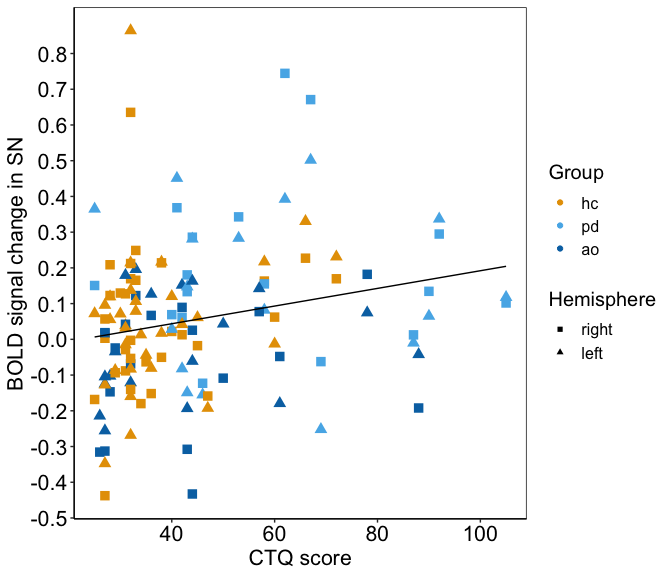


**Figure S5** Relationship between clinical variables and BOLD response to the task under placebo. Significant positive correlations were observed between BOLD response in (i) SN and (ii) VP, with CTQ and STAI-T scores respectively. HC and SD groups are denoted by yellow and blue shapes respectively. The PD and AO subgroups are further denoted by light and dark blue, and right and left hemispheres by square and triangle shapes respectively, for visual reference.


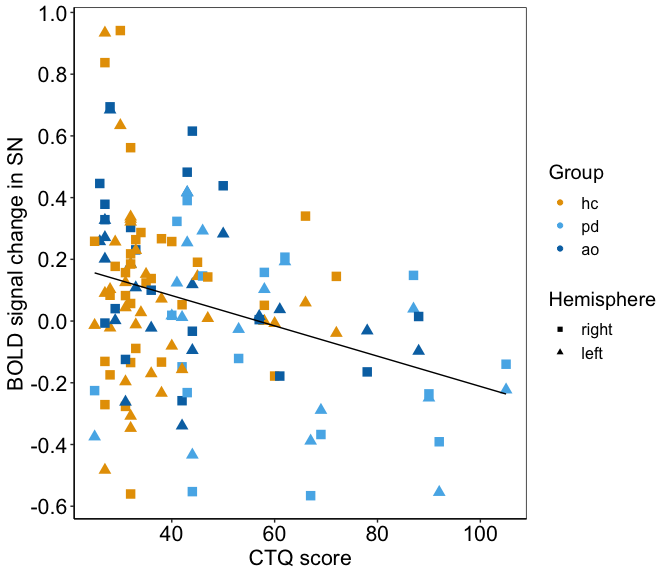

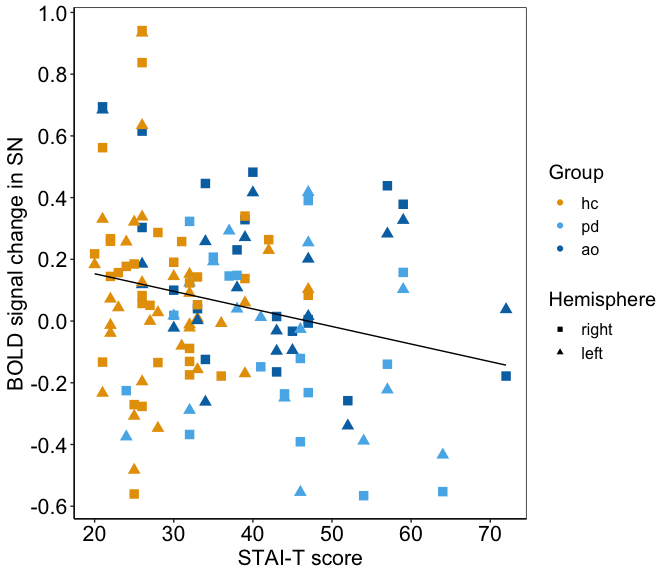

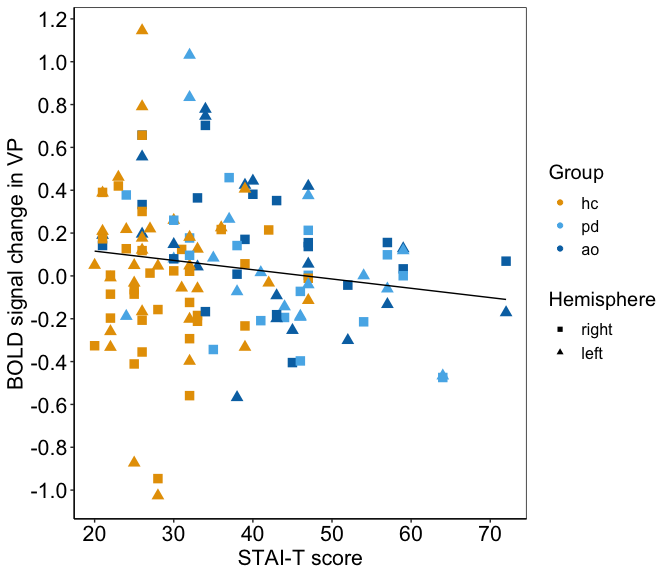


**Figure S6.** Relationship between clinical variables and BOLD response to D3 antagonism (GSK598809-placebo). Significant negative correlations were observed between BOLD response to D3 antagonism and CTQ in (i) SN and between BOLD response and STAI-T in (ii) SN and (iii) VP. Data are mean (SD) % BOLD signal change in the aversive > neutral contrast.
